# The Stereoselective Nitro-Mannich Reaction in the Synthesis of Active Pharmaceutical Ingredients and Other Biologically Active Compounds

**DOI:** 10.3389/fchem.2020.00030

**Published:** 2020-01-28

**Authors:** Ana Maria Faisca Phillips, M. Fátima C. Guedes da Silva, Armando J. L. Pombeiro

**Affiliations:** Centro de Química Estrutural, Instituto Superior Técnico, Universidade de Lisboa, Lisbon, Portugal

**Keywords:** aza-Henry reaction, imine, nitroalkane, nitroamine, piperidine, iminosugar, oxindole, phosphonate

## Abstract

The nitro-Mannich (aza-Henry) reaction, in which a nitroalkane and an imine react to form a β-nitroamine, is a versatile tool for target-oriented synthesis. Although the first stereoselective reaction was developed only 20 years ago, and enantioselective and diastereoselective versions for the synthesis of non-racemic compounds soon after, there are nowadays a variety of reliable methods which can be used for the synthesis of APIs and other biologically active substances. Hence many anticancer drugs, antivirals, antimicrobials, enzyme inhibitors and many more substances, containing C–N bonds, have been synthesized using this reaction. Several transition metal complexes and organocatalysts were shown to be compatible with the presence of a wide range of functional groups in these molecules, and very high levels of asymmetric induction have been achieved in some cases. The reaction has also been applied in cascade processes. The structural diversity of the products, ranging from simple heterocycles or azabicycles to complex alkaloids, iminosugars, amino acids or diamino acids and phosphonates, shows the versatility of the nitro-Mannich reaction and its potential for future developments.

## Introduction

**Figure 1:**
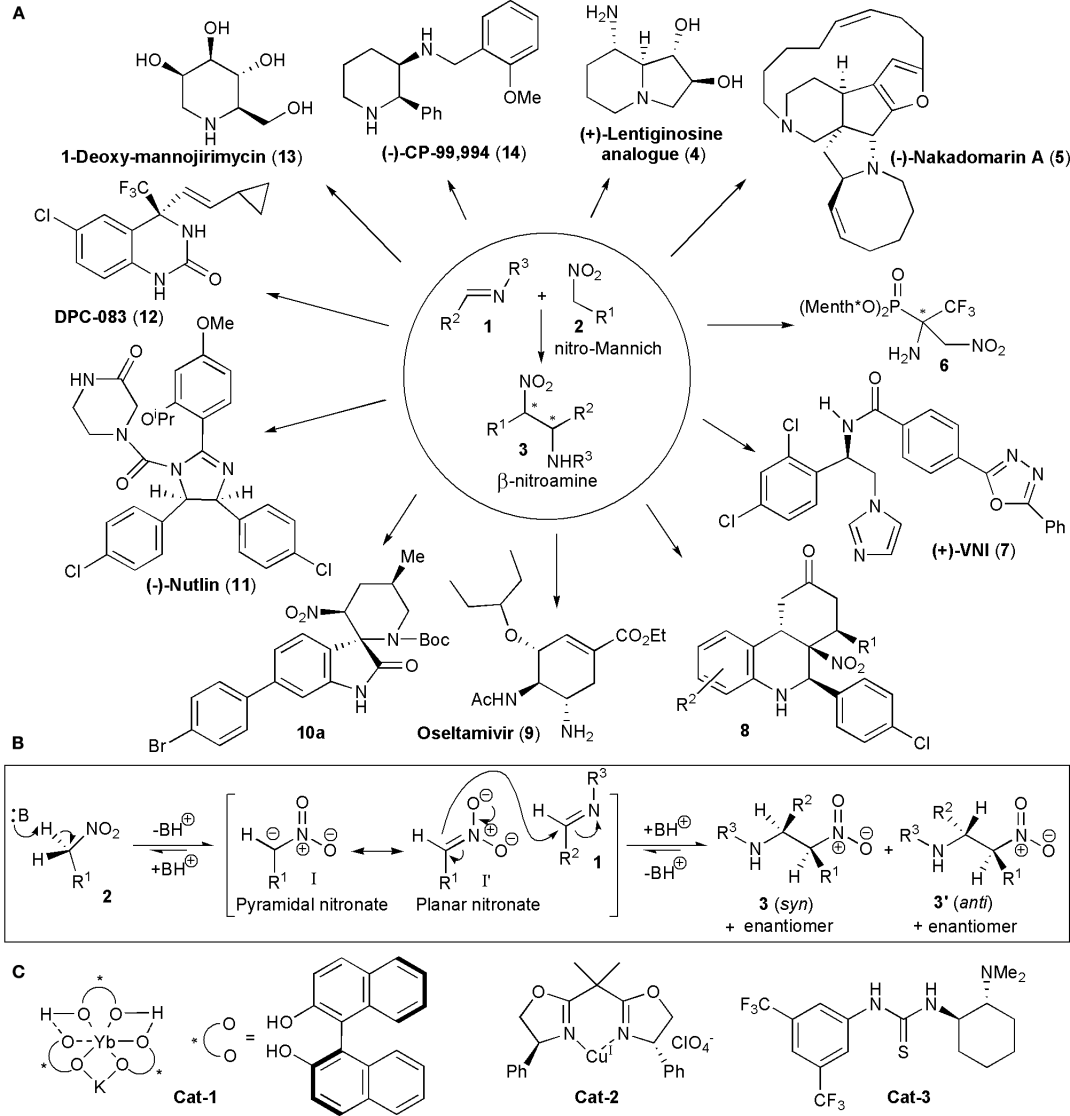
**(A)** Some of the structural diversity encountered when the nitro-Mannich reaction was utilized in the synthesis of biologically active substances or their intermediates; **(B)** the base-catalyzed nitro-Mannich reaction; **(C)** early catalysts utilized in stereoselective nitro-Mannich reactions.

The nitro-Mannich reaction, or aza-Henry reaction as it is also known, is the addition of a nitroalkane to an imine (Marqués-López et al., 2009; Noble and Anderson, 2013; Faisca Phillips, 2016; Sukhorukov et al., 2016). A carbon-carbon bond is formed and the product contains vicinal carbon-nitrogen bonds, with nitrogen in different oxidation states, which is ideal for further selective synthetic manipulations. Up to two new chiral centers can be created in the reaction. The nitro group may also be easily transformed into other functional groups, e.g., into an amine (via nitro reduction), a carbonyl group (via the Nef reaction), or it can be even be removed after performing its functions by reductive denitration. Given the high prevalence of nitrogen-containing groups present in pharmaceuticals, which are estimated to be more than 84% of the total, it is no surprise to find that the nitro-Mannich reaction has found many applications in the synthesis of natural products and synthetic biologically active substances, including existing pharmaceutical active ingredients (APIs) (Vitaku et al., 2014). Some examples of the APIs, as well as of other compounds with known biological activity (**1–14**), are shown in Figure 1A. The syntheses of these compounds are discussed later in this review. Before looking at the various strategies which make use of the nitro-Mannich reaction in target-oriented synthesis, we will consider briefly what factors have to be kept in mind when planning a synthetic route. The general mechanism for a base-catalyzed reaction is shown in Figure 1B. A pair of diastereoisomers, **3** and **3'**, may be produced, each as a racemic mixture, in the absence of a chiral influence. The nitro-Mannich reaction is facilitated by the presence of electron-withdrawing or activating groups on the imine, which increase the polarizability of the C=N double bond. Electron-withdrawing *N*-protecting groups are often used, e.g., sulfinyl, sulfonyl, phosphinoyl, and carbamoyl. However, electron-rich imines may also be employed, particularly when suitable catalysts are used, or in multi-step processes, e.g., in cascade reactions, in which the imine becomes electron deficient during the course of the reaction. When alkyl substituted imines are employed there is the possibility of facile imine-enamine tautomerization. If unstable imines are required, they may often be generated *in situ*. In the nitro-Mannich reaction all the steps are reversible and in some cases the products are reduced prior to isolation to avoid retro-addition. Problems may also arise when the β-nitroamine products are unstable in presence of base, which may be required to generate the nucleophilic nitronate anion (Anderson et al., 2005), in which case further derivatizations have to be promptly used too.

The nitro-Mannich reaction only acquired a significant role in synthetic applications in recent years, particularly when the first acyclic diastereoselective reactions were reported by Adams et al. (1998). By 1999, Shibasaki and coworkers used heterobimetallic complexes prepared from Yb(O*i*Pr)_3_, KO*t*Bu and BINOL, e.g., YbKH_2_[*(R)*-binaphthoxide]_3_, containing both Lewis-acidic and Brønsted-basic sites, to promote the reaction between nitromethane and a variety of *N*-phosphinoyl-aryl imines with good enantioselectivities, describing in this way the first example of a catalytic enantioselective nitro-Mannich reaction (**Cat-1**, Figure 1C; Yamada et al., 1999). The Shibasaki group extended soon after the scope of the reaction to nitroalkanes with longer alkyl chains, using a second generation heterobimetallic complex, i.e., a combination of Al-Li[*(R)*-binaphthoxide]_2_ and KO-*t*-Bu and described the enantioselective and diastereoselective catalytic nitro-Mannich reaction in 2001 (Yamada et al., 2001). In the same year Jørgensen and coworkers showed that by using preformed nitronates, in this case silyl nitronates, with *N*-protected α-imino esters, the use of base was unnecessary (Knudsen et al., 2001). The reaction was catalyzed by copper-bisoxazoline catalysts, e.g., **Cat-2**, and the products were obtained in a highly enantio- and diastereoselective manner. The authors also showed that the reaction products could be converted into optically active and synthetically valuable α,β-diamino acid derivatives.

In 2004 Takemoto and coworkers used a bifunctional catalyst containing both a thiourea function and a tertiary amino group (**Cat-3**) to promote the direct reaction of *N*-protected imines with nitromethane which afforded the products with high yields and enantioselectivities. He described in this way the first example of an organocatalyzed nitro-Mannich reaction (Okino et al., 2004). In the majority of nitro-Mannich reactions know so far, anti-diastereoselectivity is observed, but there are exceptions.

Earlier this year the first enzyme-promoted aza-Henry reaction was described, the addition of nitromethane to *N-*arenesulfonylaldimines, catalyzed by either lipase TL from *Pseudomonas stutzeri*, oxynitrilase from *Arabidopsis thaliana* (AtHNL) or porcine pancrease lipase (PPL) (Ignacy et al., 2019). These reactions provided high yields of products (up to 81%) but interestingly they were not stereoselective. However, further refinements may lead into a new promising area of research.

This review aims to highlight applications of the nitro-Mannich reaction in the synthesis of biologically active substances, including known APIs. It is concentrated mostly on the last four years (2016–2019), although a few previous examples are also included so that a more comprehensive view can be conveyed. The information is arranged according to the main structural element in the bioactive molecule containing either one or both C–N bonds involved in the nitro-Mannich reaction, that is:

1. Introduction
2. Piperidines and piperidinones
3. Imino sugars and other carbohydrate derivatives
4. Piperazinones, quinazolines, and related substances
5. Five membered-rings containing one or more heteroatoms
6. Oxindoles and indolenines
7. Quinolizines, dihydroisoquinolines and other azabicycles
8. Larger fused ring systems containing a shared nitrogen
9. Aminophosphonic and amino acid derivatives
10. Miscellaneous
11. Conclusions.

## Piperidines and Piperidinones

**Figure 2:**
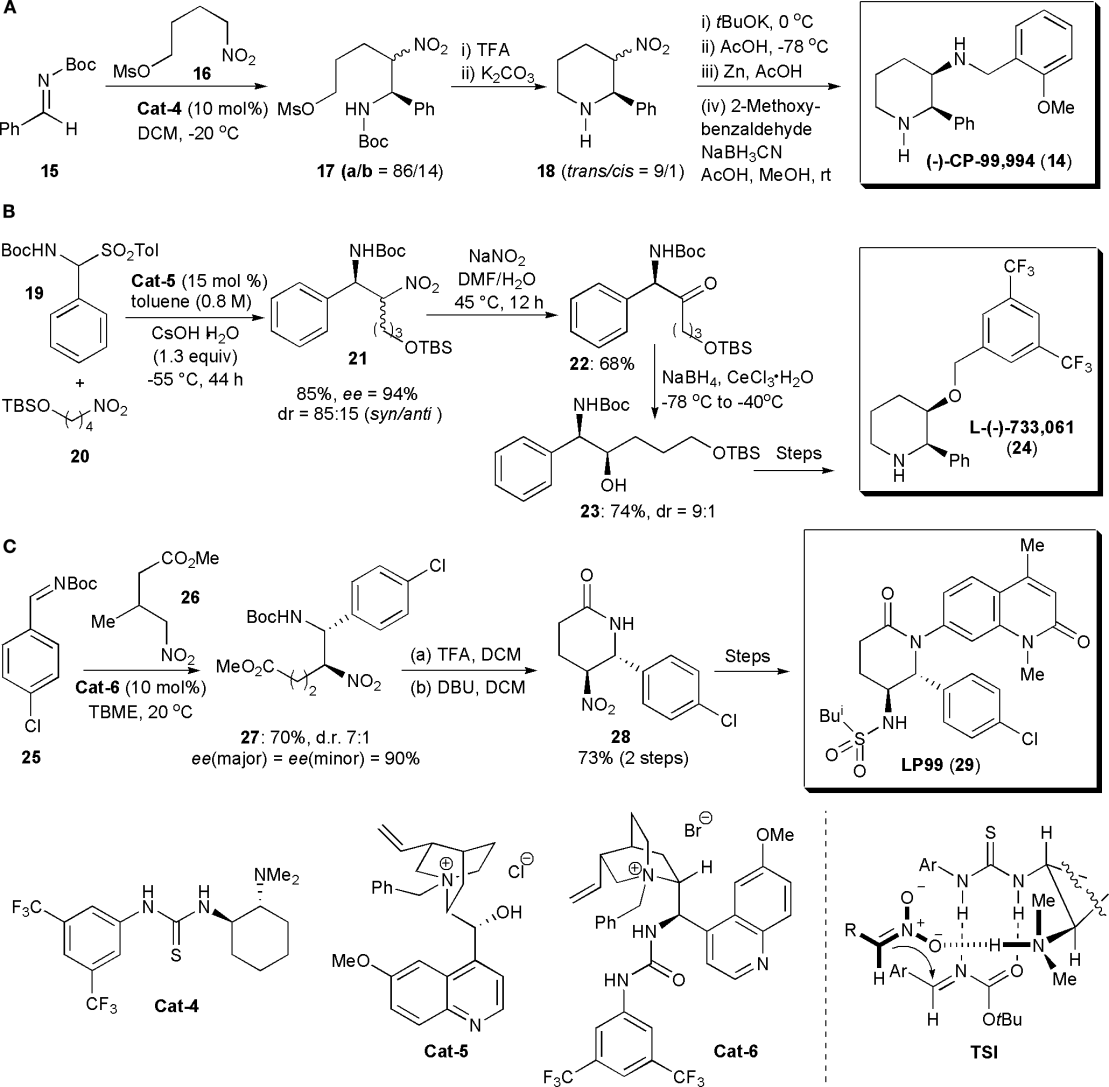
Synthesis of piperidine- and piperidinone-based drugs via a nitro-Mannich reaction: **(A)** the synthesis of CP-99,994; **(B)** the synthesis of l-(–)-733,061; **(C)** the synthesis of LP99.

Polysubstituted piperidine rings were the most common structural elements of APIs assembled via nitro-Mannich reactions encountered in the literature. This reaction was usually used in these examples to introduce the chiral centers which ultimately controlled the stereochemistry of the final products. The first application of an organocatalytic nitro-Mannich reaction to synthesize piperidines was described by Xu et al. (2006). They showed that *syn*-β-nitroamines could be obtained from nitroalkanes and *N*-Boc-imines with good to high diastereo- and enantioselectivity when these substances were allowed to react in the presence of a chiral thiourea. To account for the stereochemistry observed, the authors proposed that the reaction proceeded via the formation of complex **TSI**, in which the imine and a nitronate generated by nitroalkane deprotonation by the catalyst were brought into close proximity, in the proper orientation to react, through the formation of hydrogen bonds with the catalyst (Figure 2A). This chemistry was then used to synthesize the selective neurokinin-1 (NK-1) receptor antagonist CP-99,994 (**14**), which due to its unique biological activity, has been the subject of several studies aimed at exploring its therapeutic potential. Hence *N*-Boc imine **15** was reacted with mesylate **16**, prepared from a known nitroalcohol, in the presence of the thiourea catalyst **Cat-4**, giving rise to aza-Henry adducts **17a** and **17b** as a mixture of diastereoisomers. These isomers were not separated but cyclized with mild base after removal of the *N*-protecting group with TFA, to afford piperidines **18** as a 9/1 mixture of *trans* and *cis* isomers in 80% yield. The diastereoisomers **18** were then epimerized by a kinetically controlled protonation, after successive treatment with *t*BuOK in THF at 0°C and AcOH at −78°C, yielding the desired *cis*-isomer as the major product (*cis/trans* = 95/5). To avoid any back isomerization, the crude mixture was reduced with zinc in AcOH, and the resulting amine was combined with 2-methoxy-benzaldehyde under reductive amination conditions, to yield CP-99,994 in 75% yield (from **18**).

In 2011 Kumaraswamy and Pitchaiah developed a synthetic strategy to prepare l-(–)-733,061 (**24**), a chiral piperidine with potent neurokinin-1 (NK-1) receptor antagonist activity (Kumaraswamy and Pitchaiah, 2011a; Cochi et al., 2012). NK-1 receptor antagonists are of interest as antidepressants or even as medicines for the prevention of nausea and vomiting associated with cancer chemotherapy (Watanabe et al., 2008). Although the stereochemistry of l-(–)-733,061 was established at the start of the synthetic sequence, by an organocatalytic *syn*-selective nitro-Mannich reaction, the original nitro group was not retained in the product. Thus *N*-Boc-sulfone **19** and nitro compound **20** were reacted in the presence of chiral phase transfer catalyst **Cat-5**, using conditions previously reported for this transformation (Palomo et al., 2005) affording nitroamine **21** in high yield (85%), dr (*syn*/*anti* = 85:15) and enantioselectivity [*ee*(*syn*) = 94%] (Figure 2B). Oxidation of the nitro group of the diastereoisomers with Gissot's protocol gave rise to amino ketone **22**. Subsequently the ketone could be reduced with NaBH_4_ in the presence of CeCl_3_·7H_2_O to afford secondary amino alcohol **23** in a *syn* selective manner (dr = 9:1) and high yield (74%). The configuration of the new chiral center is controlled by the configuration of the adjacent carbon atom bearing the amino group, established initially in the nitro-Mannich reaction. Hydroxyl group protection, TBS deprotection, mesitylation, *N*-Boc deprotection and finally cyclization under basic conditions, afforded piperidine l-(-)-733,061 (**24**), in 65% isolated yield (over the last three steps).

The piperidinone ring (a lactam) is another motif frequently encountered in biologically active substances. Brennan, Dixon, and coworkers developed nitro-Mannich/lactamization cascade processes to synthesize a series of quinolone-fused lactams for early structure–activity relationship studies in a search for bromodomain inhibitors (Clark et al., 2015). The bromodomain (BrD) is a protein domain responsible for the recognition of acetylated lysine residues in histones and nuclear proteins in regulation of gene transcription in chromatin (Ren et al., 2016). Many proteins have BrDs which, if deleted or forced to mutate, lead to an impairment of the protein function. Hence they play a critical role in human biology and disease. Both BRD7 and BRD9 are often overexpressed in cancer (Clark et al., 2015). Since their interactions with chromatin are complex, selective, potent inhibitors of these bromodomains could be valuable tools. Until this report, there were no records describing selective inhibitors of BRD7 and BRD9. A series of potential inhibitors was generated, based on biophysical assays. A valerolactam unit was identified as the most promising for further investigations. Several nitro-substituted lactams were synthesized, with the nitro group being used for further derivatizations. LP99 was identified as the best inhibitor. A stepwise approach was used to obtain this molecule. Its synthesis started with an enantioselective nitro-Mannich reaction catalyzed by quinidine-urea **Cat-6**. Product **27** was obtained on a gram scale as a 7:1 mixture of diastereomers, both in 90% *ee*. *N*-Boc deprotection and concomitant cyclization with TFA, followed by epimerization with DBU, afforded lactam **28** as a single diastereomer in high yield (Figure 2C). The quinolone moiety could be introduced by Cu-mediated Goldberg coupling (7-bromo-1,4-dimethyl-1*H*-quinolin-2-one, copper iodide, potassium phosphate, *trans*-cyclohexyl-1-2-diamine, in dioxane) after reduction of the nitro group and *N*-Boc protection of the resulting amine (not shown). Finally deprotection of the amine and reaction with *iso*butanesulfonyl chloride led to LP99, the most potent of all the analogs prepared, in 90% *ee* (>99% after HPLC purification). LP99 was shown to inhibit the association of BRD7 and BRD9 to acetylated histones *in vitro* and in cells and it was also demonstrated that BRD7/9 plays a role in regulating pro-inflammatory cytokine secretion.

**Figure 3:**
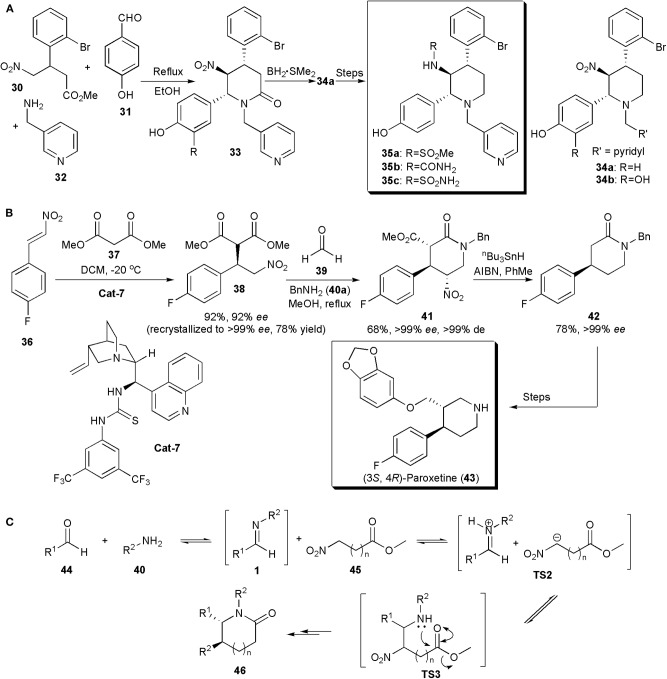
**(A)** Synthesis of piperidine-based compounds 17 via multicomponent reactions; **(B)** the synthesis (3*S*,4*R*)-Paroxetine via an enantio- and diastereoselective nitro-Mannich/lactamization cascade reaction; **(C)** the mechanism proposed for the nitro-Mannich/lactamization cascade reaction (Pelletier et al., 2009).

Multi-component reactions have also been used to assemble the piperidine ring of APIs and other biologically active compounds. This was the case with a protein farnesyltransferase (FTase) inhibitor, described by Tanaka et al. (2007). FTase plays a crucial role in the signal transduction pathway. Some FTase inhibitors are being studied as promising anti-cancer drugs, as for example, the nitro piperidine derivatives **34** (Figure 3A). However, although compounds **34** have potent FTase inhibitory activity (**34a**, IC_50_ = 5.4 nM and **34b**, IC_50_ = 3.7 nM), they are rapidly cleared, which limits their application. Pharmacokinetic studies suggested that glucuronidation of the C-2 phenolic group could be the factor responsible for the fast clearance. Aiming to improve the metabolic stability of the compounds, Kanda and coworkers developed a new series of piperidines with additional modifications. Substituents were introduced at the position *ortho* to the phenolic hydroxyl group, aiming to block glucuronidation by an increase in adjacent steric bulk. The piperidine core was assembled via a method previously developed, a three-component reaction between a 4-nitrobutyrate, a benzaldehyde and an amine, which yielded the desired product containing a 1,2-dinitrogen substituted fragment in a highly diastereoselective manner, in quantitative yields (Figure 3A). No catalyst was required. The amide linkage initially produced could be reduced with the borane-methyl sulfide complex to give piperidine **34a** in 62% yield. Only one diastereoisomer was observed in all cases. By varying the substitution patterns of these starting materials several piperidines could be obtained for structure-activity relationship studies. A 4,5-*trans*-5,6-*trans* configuration appears to be thermodynamically favorable because all the substituents are equatorial. Further synthetic manipulations provided additional analogs. It was observed that the introduction of bulky groups next to the C-2 hydroxyl did not affect potency e.g., methanesulfonylamino **35a** (IC_50_ = 4.3 nM), urea **35b** (IC_50_ = 6.2 nM), and sulfamoylamino **35c** (IC_50_ = 3.0 nM) groups, but in addition the compounds produced also showed good metabolic stability *in vitro*. Other combinations were possible. The studies also showed that the configuration of the chiral centers was important for activity. Hence chiral resolution showed that whereas (+)-**35b** was highly active, its (–)-enantiomer, i.e., (–)-**35b**, was a weak FTase inhibitor (IC_50_ > 310 nM) (Nara et al., 2003).

In the synthesis of (3*S*,4*R*)-Paroxetine, developed by Dixon and coworkers in 2008 (Figure 3B), the key step in which the stereochemistry of the product is introduced is not a nitro-Mannich reaction but a highly enantioselective Michael addition, in which dimethyl malonate adds to 1-fluoro-4-(2-nitro-vinyl)-benzene under the influence of a bifunctional Lewis base, Brønsted acid organocatalyst derived from 9-amino(9-deoxy) epicinchonine **(Cat-7)** (Hynes et al., 2008; Jakubec et al., 2012a). The nitroalkane produced is further enantiomerically enriched by recrystallization, and submitted to a three-component reaction similar to that in the example above, yet asymmetric, i.e. a nitro-Mannich lactamization cascade with the imine generated *in situ* from a reaction between formaldehyde and benzylamine, to produce **41** in an excellent enantio- and diastereoselective fashion (>99% *ee*, >99% *de*) in 68% yield. Concomitant removal of the nitro group and dealkyl decarboxylation of piperidinone **41** with a mixture of tributyltin hydride and AIBN in toluene generated **42**, previously shown to be an intermediate for the synthesis of (3*S*,4*R*)-Paroxetine (**43**), a selective serotonin reuptake inhibitor used in the treatment of depression. Hence the new organocatalytic asymmetric synthesis of **42** constitutes a formal total synthesis of (3*S*,4*R*)-Paroxetine. A general mechanism for the nitro-Mannich lactamization cascade, exemplified for piperidinones, is shown in Figure 3C (Pelletier et al., 2009). An aldehyde (**44**) is reacted with an amine (**45**) to generate an imine of type **1**, which reacts with the nitroester at the most acidic site generating a salt (**TS2**). Addition of the nitronate to the imine creates **TS3**, which undergoes lactamization to afford the piperidinone (**46**).

## Imino Sugars and Other Carbohydrate Derivatives

Imino sugars (azasugars) are amongst the most studied carbohydrate mimetics (Harit and Ramesh, 2016). They are analogs of pyranoses in which the endocyclic oxygen atom has been replaced by nitrogen. In addition, the anomeric hydroxyl group is absent. Most iminosugars are inhibitors of the corresponding glycosidases, and since these enzymes are involved in several metabolic pathways, imino sugars have also been found to display several types of biological activities, such as anticancer, antiviral (including AIDS) and even useful properties for the treatment of diabetes. Small changes of stereochemistry or structure, e.g., functional group variations, alter their potency and specificity.

**Figure 4:**
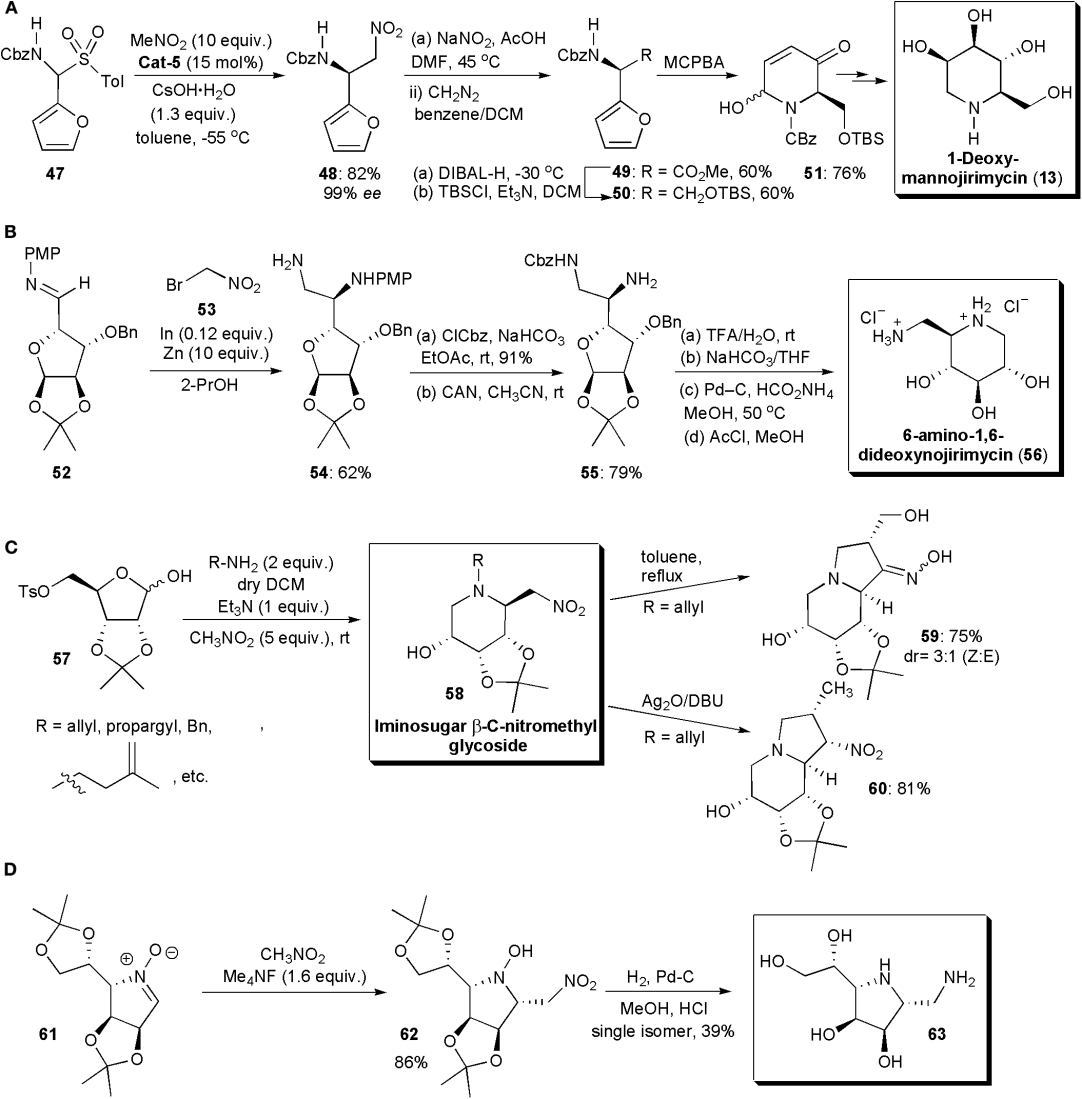
Synthesis of iminosugar-based drug intermediates and related compounds: **(A)** the synthesis of a d-deoxymannojirimycin intermediate; **(B)** the synthesis of 6-amino-1,6-dideoxynojirimycin; **(C)** the synthesis of bicyclic imino sugars **59** and **60**; **(D)** the synthesis of **63**.

A few imino sugars have been synthesized in recent years by stereoselective nitro-Mannich reactions. In 2011 Kumaraswamy and Pitchaiah described a highly enantioselective synthesis of furyl-substituted (benzyloxy)carbonyl (Cbz) protected diamino acid esters through a catalytic phase-transfer aza-Henry reaction (Figure 4A; Kumaraswamy and Pitchaiah, 2011b). These protected nonproteinogenic α,β-diamino acids (DAPS), are of interest, since when incorporated into peptides, they can modulate their secondary and tertiary conformations; the peptides also become resistant to proteolysis (Viso et al., 2005; Arrayás and Carretero, 2009). Compounds derived from them often display a wide range of biological activities. For the reacting amine, *N*-Cbz-furylamine (**47**) was selected. Masking the carboxylic acid as a furan enhances its solubility. In the presence of quininium **Cat-5** and CsOH·H_2_O, the nitro-Mannich reaction with nitromethane performed at −55 °C, gave rise to the desired product **48** in high yield and *ee* (85% yield, 94% *ee*). When β-nitroamine **21** was subjected to oxidation under Nef conditions (NaNO_2_/DMF) the corresponding acid was obtained, which was subsequently esterified to yield **49**. After reduction with DIBAL-H (diisobutylaluminium hydride) and protection of the primary alcohol with (*tert*-butyl)dimethylsilyl chloride, it afforded a silyl ether derivative (**50**) which, upon exposure to an aza-Achamotowicz rearrangement, i.e., the oxidative rearrangement of the α-furanylamine into the corresponding piperidinone (Haukaas and O'Doherty, 2001; Van der Pijl et al., 2015), afforded **51**. Piperidinone **51** has been used as a key intermediate in the synthesis of several biologically active substances and pharmaceuticals, e.g., the natural product d-deoxymannojirimycin, (2*R*,3*R*,4*R*,5*R*)-2-(hydroxymethyl)piperidine-3,4,5-triol (DMJ) and its l-isomer, which are imino sugars (Haukaas and O'Doherty, 2001). Iminosugars are known to be excellent glycosidase inhibitors (Sears and Wong, 1999). DMJ in particular has been shown to be a potent inhibitor of the mammalian Golgi α-mannosidase-1 activity, blocking the conversion of high-mannose oligosaccharides to complex oligosaccharides without inhibiting the biosynthesis of lipid-linked oligosaccharides (Humphries et al., 1988). The present method for the synthesis of **51** constitutes a formal synthesis of this biologically active substance. *N*-Alkylated derivatives of 1-deoxynojirimycin, e.g., (2*R*,3*R*,4*R*,5*S*)-2-(hydroxymethyl)piperidine-3,4,5-triol (DNJ), Glyset and Zaveska, are prescribed drugs for the oral treatment of type II diabetes and Gaucher's disease, respectively. They differ from DNJ by the presence in the molecule of an *N*-ethyl alcohol and an *N*-butyl substituent, respectively, instead of the free amine.

The analog of deoxymannojirimycin, 6-amino-1,6-dideoxynojirimycin, has been frequently used in synthesis as precursor for a wide range of glycosidase inhibitors (Pampín et al., 2009; Soengas and Silva, 2013). Its asymmetric synthesis has also been described by an aza-Henry reaction catalyzed by indium (Figure 4B). Although indium has been known for a while to catalyze a variant of the aza-Henry reaction, the addition of bromonitroalkanes to sugar imines, this relatively expensive metal was used only in stoichiometric amounts. In the newly developed protocol, Soengas and Silva used catalytic amounts of indium, which was possible due to its combination with zinc dust. Zinc dust alone did not catalyze this transformation, which suggests that it does not play the role of active catalyst in the reaction, but that it serves to regenerate the active indium species during the catalytic cycle. Diamine **54** was selected as strategic intermediate. Starting from enantiomerically pure sugar imine **52**, the reaction with bromonitromethane **53** was performed in the presence of In (0.12 equiv.) and Zn dust (10 equiv.) in THF, with sonication. The aza-Henry product **54** was obtained in 62% yield. Only the *anti* isomer was observed, as expected from a Felkin–Anh antiperiplanar nucleophilic addition of the organoindium species to the imine carbonyl. Cbz protection of the primary amino group and removal of the PMP protecting group yielded diamine **55**. Once the acetonide was deprotected by treatment with acid, basic treatment and catalytic hydrogenation afforded the desired imino sugar, 6-amino-1,6-dideoxynojirimycin, as the hydrochloride salt, after exposure to methanolic HCl.

In 2017, Baskaran and coworkers reported the development of novel methods for the synthesis of iminosugar *C*-nitromethyl glycosides and bicyclic iminosugars (Prasad et al., 2017). The first group of compounds could be obtained from d-ribose tosylate **57**, by treatment with an amine at room temperature, and subsequent alkylation of the iminium ion generated *in situ* with nitromethane (Figure 4C). The iminosugars were obtained with drs varying from 1:0 to 9:1 and in good yields. The nitro group present in the molecules could be used for further synthetic manipulations. Hence, the products **58** (when R=allyl or propargyl) were converted to indolizidine derivatives, e.g., **59**, simply by heating, through a novel intramolecular cyclization, which afforded bicyclic iminosugar-oxime derivatives. Otherwise, when they were exposed to SET oxidative radical cyclization (with Ag_2_O and DBU) under Kamimura reaction conditions, a *5-exo-trig* radical cyclization took place to furnish indolizidine derivatives, e.g., **60**, in good yields and excellent stereoselectivities.

Nitrones have also been applied in nitro-Mannich reactions, although not very frequently, replacing imines or imine equivalents. These substances have a highly polarized double bond, and hence they are electrophilic in nature and susceptible to nucleophilic addition reactions. They have been used in the past as key substrates for the synthesis of iminosugars (Messire et al., 2019). In 2019 Behr and coworkers reported new studies involving nitrones (Figure 4D). A nitromethylation of nitrones with CH_3_NO_2_ was performed in the presence of tetramethylammonium fluoride or triazabicyclodecene, which were added as promoters (Messire et al., 2019). Phase transfer catalysts could also be used, which suggests that enantioselective transformations may become possible with further research. A large excess of the nitroalkane was needed to ensure high conversion. Nitroethane and nitropropane could also be used, affording mixtures of *syn* and *anti* stereoisomers with low diastereoselectivity. The products were converted into vicinal diamines by H_2_ reduction in the presence of palladium-carbon. Nitrones derived from protected imino sugars could be used as substrates, e.g., **61** and in this case the synthesis of a yet unknown diaminoimino sugar (**63**) could be performed, via aza-Mannich adduct **62**. The methodology developed was also used in the production of (+)-Praziquantel (PZQ), the main drug prescribed against all Schistosoma species and indicated in the WHO's list of essential drugs (WHO = World Health Organization). The synthesis of PZQ is discussed further in the next section of this chapter.

**Figure 5:**
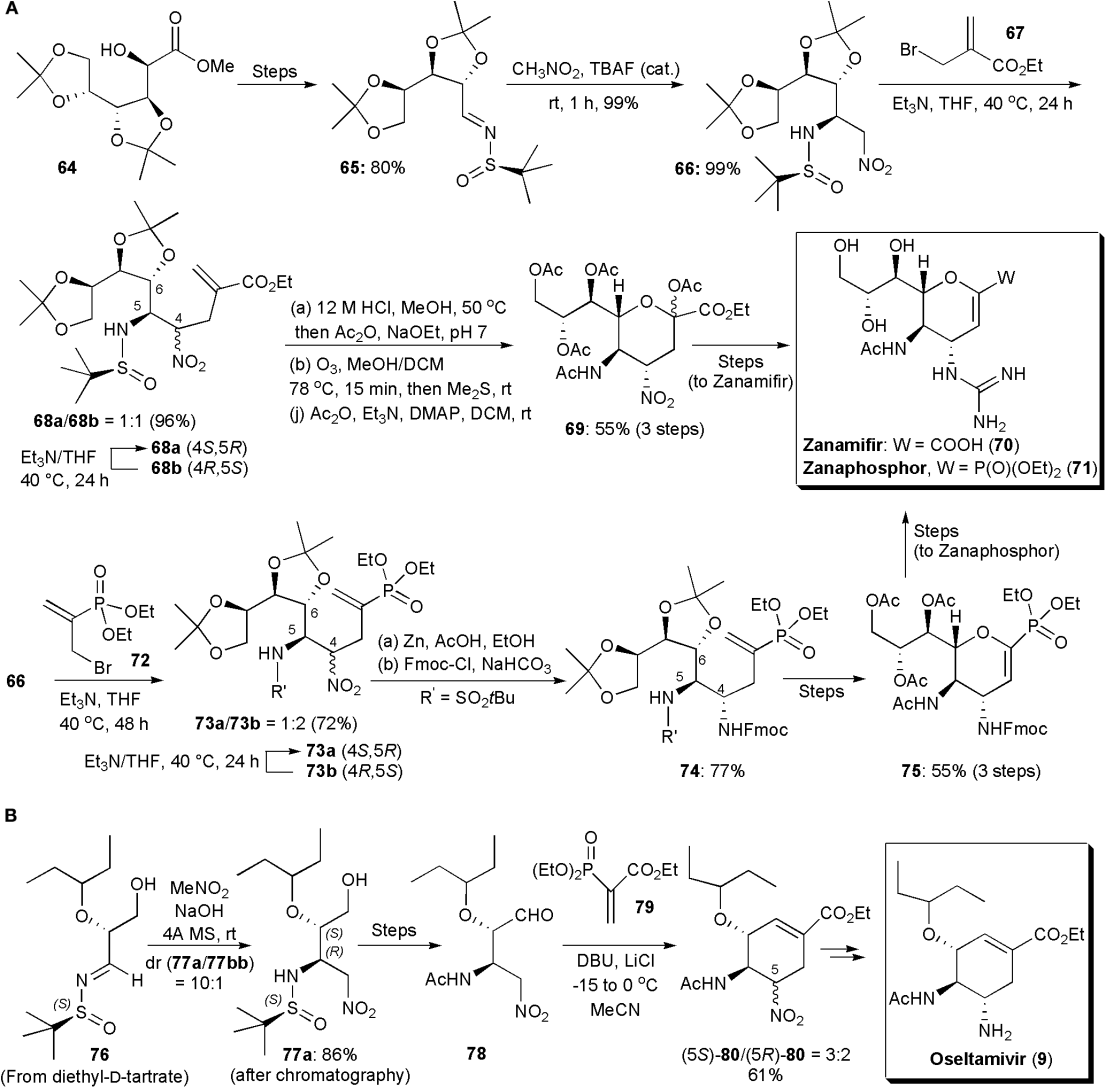
**(A)** The synthesis of sugar-based antivirals Zanamifir and Zanaphosphor and **(B)** the synthesis of Oseltamivir.

Among notorious carbohydrate-based small molecule drugs can be found the potent anti-influenza agents Zanamivir and Zanaphosphor. These substances inhibit neuraminidases, glycoside hydrolase enzymes responsible for breaking the linkage between the influenza virus and the sialo receptor of host cells, the process by which newly formed virus particles are released to infect other cells [300]. Presently oseltamivir (**9**) is the most popular anti-influenza drug, but since resistance can occur, other substances are continuously being developed to treat influenza. Both Zanamivir, as well as its phosphonate analog Zanaphosphor, have been synthesized via aza-Henry reactions, and even Oseltamivir, which is also a neuraminidase inhibitor. Zanamivir and Zanaphosphor are less prone to be affected by resistance, because they have in their structures a glycerol side chain, common to that found in the structure of sialic acid (nacetylneuraminic acid), which is essential to bind influenza hemagglutinin with host cells. The aza-Henry approach to these two substances was reported in 2016 (Lin and Fang, 2016). In this procedure (Figure 5A), an inexpensive d-glucono-δ-lactone (**64**), whose synthesis has been previously reported (Yadav et al., 2009), is reacted with a chiral sulfinylimine, to produce the substrate (**65**) for the aza-Henry reaction with nitromethane. The new nitro group is used as a latent amino group. The aza-Henry reaction places the two nitrogen-containing substituents at the C4 and C5 positions in the desired absolute configuration. This step avoids the use of a hazardous azide reagent. Tetrabutylammonium fluoride was used to catalyze the aza-Henry reaction, which proceeded well affording 99% yield of the addition product **66** exclusively in the (5*R*)-configuration, as confirmed by X-ray diffraction analysis. This remarkable stereoselectivity was caused by the use of the (*R*)-*tert*-butylsulfinamide group as chiral auxiliary. To extend the carbon chain the sulfonamide was alkylated with ethyl 2-(bromomethyl)acrylate (**67**) in the presence of excess Et_3_N, affording 96% of a 1:1 mixture of **68a** and **68b**. Diastereoisomer **68a** could be separated from its epimer **68b** by a simple extraction with *n*-hexane, and epimer **68b**, which was the thermodynamically least stable, could be converted into **68a** by treatment with Et_3_N for a prolongued period. Hydrolysis of the acetal and sulfinylimine groups, protection of the amine by acetylation, and ozonolysis, gave an α-oxo ester, which underwent an intramolecular nucleophilic attack by the C6 hydroxyl group to form the tetrahydropyran ring which led to **69** in 55% yield. After a few additional synthetic steps Zanamivir (**70**) was obtained, its total synthesis requiring 12 steps overall. The aza-Henry intermediate **66** was used as the key synthetic intermediate for the production of Zanaphosphor. The phosphonyl group was introduced by the treatment of **66** with diethyl (3-bromoprop-1-en-2-yl)phosphonate (**72**) and Et_3_N. The resulting epimers (**73a**/**73b**) could be separated in a manner similar to that used for Zanamivir, and the thermodynamically least stable epimer was recycled in the same way. The remaining synthesis had to be modified, since after ozonolysis of the double bond the presumed α-oxophosphonate which would be produced to lead to the tetrahydropyran, had both a hydroxyl and a phosphonate groups at the C2 position, which made it unstable in both acids and bases. Hence a different approach had to be followed. The use of a Fmoc protecting group after reduction of the nitro group resolved the problem. After ozonolysis of the double bond in **74**, and tetrahydropyran formation, peracetylation was performed using iodine as a Lewis acid catalyst. The dihydropyran **75** was obtained presumably as a result of *in situ* elimination. Zanaphosphor (**76**) could be produced smoothly from this intermediate (Lin and Fang, 2016).

The synthesis of oseltamivir itself via an aza-Henry reaction was accomplished already in 2010, by Lu and coworkers (Figure 5B; Weng et al., 2010). The total synthesis of this carbocycle, starting from diethyl d-tartrate, an inexpensive and abundant starting material, involved 11 steps. The asymmetric aza-Henry reaction and a domino nitro-Michael/Horner-Wadsworth-Emmons (HWE) reaction were key steps to construct the cyclohexene ring of the product. The use of an enantiopure sulfoxide *N*-protecting auxiliary, instead of a Boc protecting group, allowed better enantiocontrol, from 5:1 to 10:1 with the major diastereoisomer of **77**, i.e., **77a**, having the same absolute and relative configuration at the two stereogenic centers as that of oseltamivir. The isomers could be separated by column chromatography. The cyclohexenecarboxylate skeleton was assembled after removal of the *tert*-butylsulfinamide protecting group, acetylation of the resulting amine and oxidation of the primary alcohol to an aldehyde (not shown in the Scheme). A Michael addition/intramolecular HWE domino reaction upon treatment of **78** with vinylphosphonate, produced cyclohexene **80** as a 3:2 diastereoisomeric mixture in 61% yield. The major isomer had the desired configuration and it could be converted directly into oseltamivir (**9**), by reduction with Zn/AcOH, in 71% yield.

## Piperazinones, Quinazolines and Related Substances

**Figure 6:**
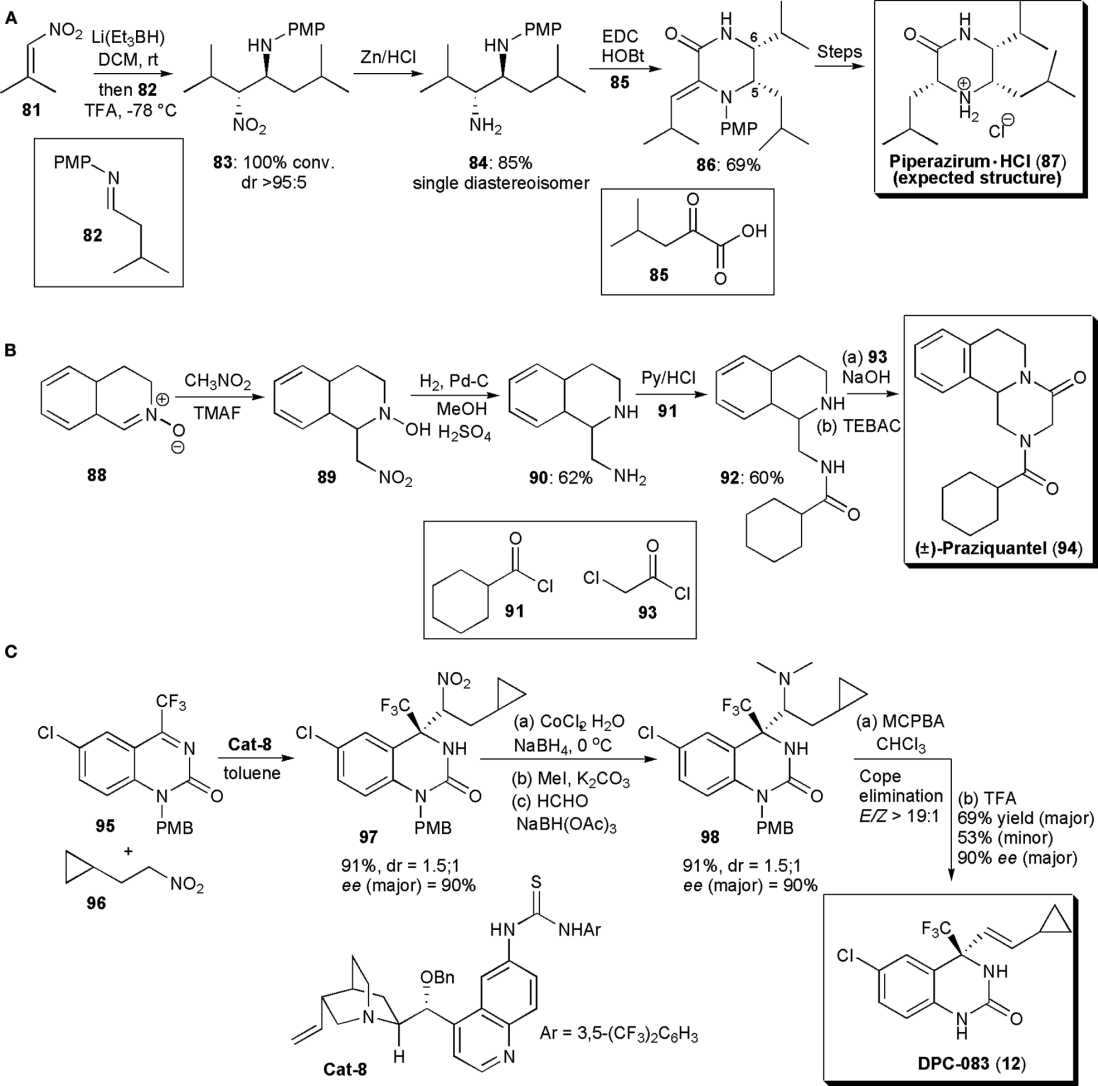
**(A)** The synthesis of Piperizirum. HCl; **(B)** the synthesis of (±) -Praziquantel; **(C)** the synthesis of DPC-083.

Piperazirum is an alkaloid isolated from a leaf extract of *Arum palaestinum* Boiss, which displays significant cytotoxicity against cultured tumor cell lines *in vitro*. Anderson and coworkers developed a method to produce a synthetic sample to help elucidate its stereochemistry (Figure 6A; Anderson et al., 2013). A reductive nitro-Mannich reaction was used to assemble a 1,2-diamine fragment of the molecule (**83**), thus establishing the C-5/C-6 relative stereochemistry. Hence, conjugate addition of hydride to a nitroolefin generated the nitronate anion which was trapped by imine **82**. The existing knowledge on this reaction shows that in the majority of cases the products of this reaction have *anti*-relative stereochemistry. To check if this was indeed the outcome of the reaction, the nitro group was reduced with Zn/HCl and the resulting diamine (**84**) was purified by column chromatography. As a single diastereoisomer, **84** was treated with thiophosgene to afford an imidazolidine-2-thione, whose stereochemistry was confirmed to be *cis* by NOE studies, a fact which confirmed that the precursor had an *anti*-relative stereochemistry. Diamine **84** was reacted with keto acid derivative **85**, in the presence of EDC (1.50 equiv) and 1-hydroxybenzotriazole (1.50 equiv) at rt, to afford the desired piperazinone **86** in good yield. A *Z*-geometry was inferred from NOESY ^1^H NMR spectroscopy. Reduction of the double bond (with H_2_, Pd-C) proceeded in a stereoselective fashion, with the stereochemistry of the new chiral center at C3 being controlled by the two existing chiral centers. *N*-deprotection with CAN and salt formation afforded a structure previously described as piperazirum (**87**). The structure of the product of these reactions was finally deduced from spectroscopic studies and confirmed by an X-ray crystallographic analysis. Although this was indeed the structure expected, according to the existing literature, the results obtained in this study did not agreed with those known for the natural product, and suggest that the configuration assigned originally to the alkaloid is incorrect and that new studies are required to determine the correct configuration.

During their studies with nitrones, described in the previous section, Behr and coworkers developed a method for the synthesis of (+)-Praziquantel (PZQ) (**94**), as mentioned above (Messire et al., 2019). Starting from nitrone **88**, generated from 3,4-dihydroisoquinoline, the aza-Mannich reaction with nitromethane led to hydroxylamine **89** in high yield (Figure 6B). Reduction of the nitro group and of the hydroxylamine (H_2_, Pd-C) to **90**, followed by regioselective acylation with **91** under known conditions afforded **93** in good yield. To end, cyclization with chloroacetyl chloride under phase transfer catalysis using benzyltriethyammonium chloride afforded Praziquantel (**94**) in 56% overall yield.

Until 2011, the available methodology to perform catalytic asymmetric aza-Henry reactions was restricted to the reactions of imines derived from aldehydes and nitroalkanes, and only one example was known in which acyclic ketimines were used as starting materials, from Feng and coworkers, who used catalysis with a chiral *N,N'*-dioxide copper complex (Tan et al., 2008). This issue was further addressed by Wang and coworkers, who found that a hydrogen-bonding catalyst, a thiourea derived from quinine, could be used to promote a highly enantioselective addition of nitroalkanes to cyclic ketimines, under mild reaction conditions, in high yields and with a catalyst loading as low as 1 mol% (Xie et al., 2011). The new method (Figure 6C) was applied to the synthesis of drug candidate DPC 083, a dihydroquinazolinone bearing a chiral trifluoromethyl moiety, which is a potent HIV-1 nonnucleoside reverse transcriptase inhibitor. Trifluoromethylquinazolin-2(1*H*)-one **95** was reacted with nitroalkane **96** under the optimized conditions, affording the corresponding nitro-Mannich product **97** in high yield (91%) and enantioselectivity [*ee*(major) = 90%]. Two diastereoisomers were obtained in this reaction, in a ratio of 1.5:1, and the remaining reaction steps were applied to the diastereoisomeric mixture. Reduction of the nitro group and demethylation afforded **98** in high yield and stereoselectivity. Oxidation of the tertiary amine led to Cope elimination, establishing the double bond with a 19:1 stereoselectivity (*E/Z*). Finally, upon PMB deprotection with TFA, product (**12**) was obtained as a mixture of diastereoisomers in good yield (69% major, 53% minor) and very high *ee* (90% major, 95% minor). No racemization of the quaternary stereocenter was observed during the synthesis of DPC under the conditions used.

## Five Membered-Rings Containing One Or More Heteroatoms

**Figure 7:**
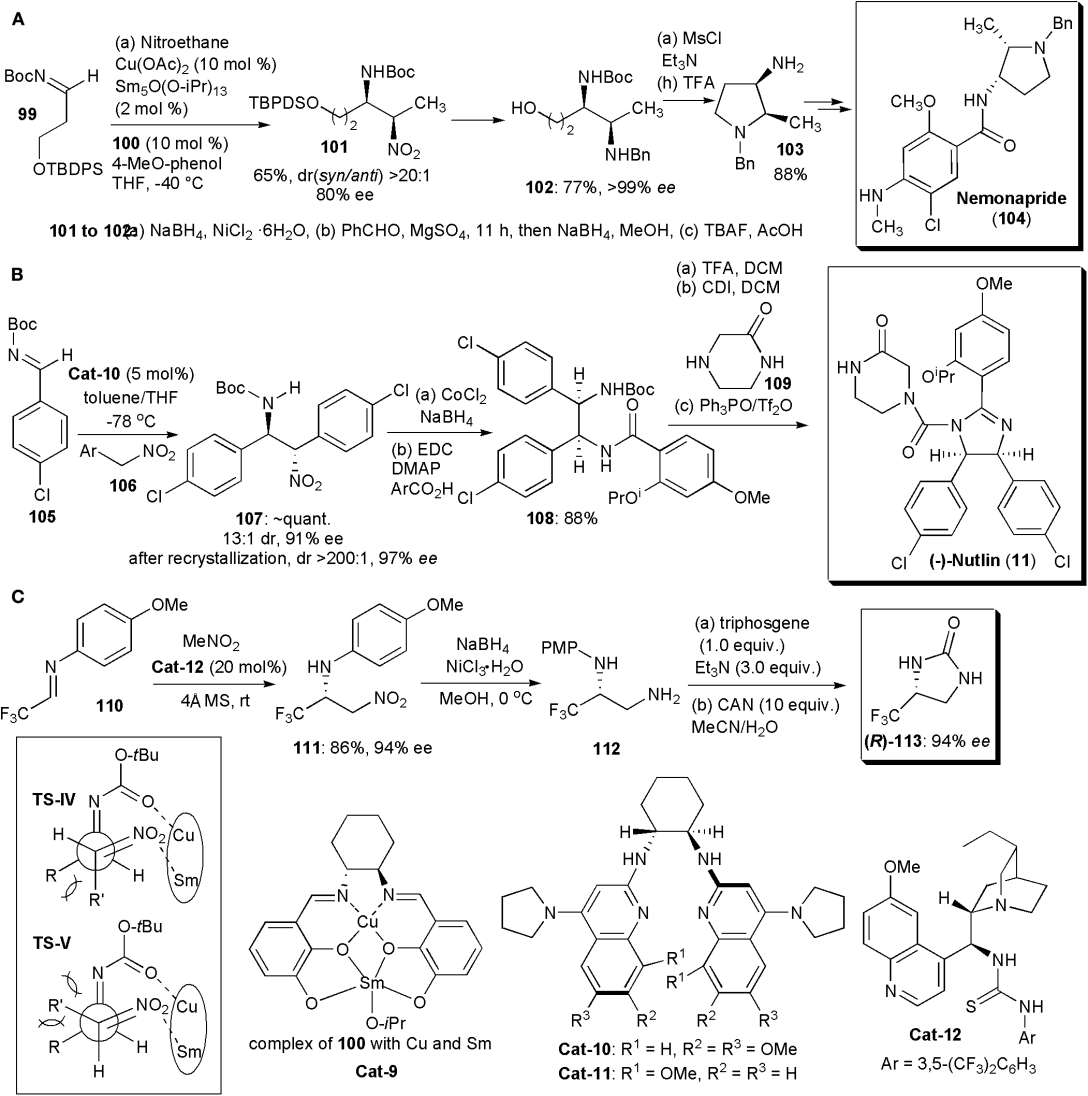
Synthesis of APIs and intermediates with five-membered *N*-heterocyclic cores: **(A)** the synthesis of Nemonapride; **(B)** the synthesis of (–)-Nutlin; **(C)** the synthesis of fluorinated 2-imidazolidone (*R*)-**113**.

Matsunaga and Shibasaki continued to pursue their interest in chiral heterobimetallic complexes which could behave as bifunctional catalysts, activating both nucleophiles and electrophiles. Using combinations of a transition metal, a rare earth metal and a chiral Schiff base ligand capable of forming dinuclear complexes, they developed the first examples of catalytic asymmetric *syn-*selective nitro-Mannich reactions in 2010 (Figure 7A; Handa et al., 2010). A combination of copper acetate, samarium isopropoxide, and ligand **100**, used in a 1:1:1 ratio, were found to be the best catalyst combination, particularly in the presence of 4-*t*Bu-phenol as additive. The catalyst promoted the nitro-Mannich reaction between *N*-Boc imine **99** and nitroethane, yielding the product in 96% yield, dr (*syn/anti*) >20:1 and *ee*(*syn*) = 94%. Several other amines and nitroalkanes were compatible with this reaction, yielding products in very high yields, drs and *ees*. The authors also applied the new methodology to the synthesis of Nemonapride (**104**), used clinically as an antipsychotic agent. The *syn*-selective nitro-Mannich reaction was used to create the chiral centers of the pyrrolidine ring (Figure 7A). Key intermediate **101** was produced via the nitro-Mannich reaction with high dr and *ee*. Subsequently the nitro group of **101** was reduced and the resulting amine benzylated by reductive alkylation. Once the TBDPS protecting group of the primary alcohol was removed, product **102** could be cyclized to yield **103**, which was purified by recrystallization (77% yield, >99% *ee*). The cyclization to the pyrrolidine was performed by treating **103** with MsCl in the presence of triethylamine, which promoted an intramolecular nucleophilic attack by the amine with displacement of OMs. Pyrrolidine **103** was obtained in 88% yield and subsequently deprotected and condensed with the necessary mixed anhydride to give Nemonapride (**104**) in 74% yield over two steps. The nitro-Mannich reaction is thought to proceed via a transition state **TS-IV**, less hindered than **TS-V**. In these transition states Sm and Cu are complexed to the catalyst. Sm, acting as a Brønsted base, generates a Sm-nitronate and Cu(II), acting as a Lewis acid, controls the position of the *N*-Boc-imine, In this way the two reacting partners are brought together in a favorable orientation to react, yielding the *syn* product.

In 2011, Davis and Johnston found a way to overcome the usual low enantio- and diastereoselection (≤2:1) usually observed in catalytic aza-Henry reactions of aryl nitromethanes, which is in contrast to the otherwise high stereoselection which can be obtained with other nitroalkanes (Davis and Johnston, 2011; Noble and Anderson, 2013; Faisca Phillips, 2016). The key was the use of an electron rich chiral bis(amidine) as catalyst. Optimization of the bis(amidine) structure revealed **Cat-10** as the best catalyst, which was subsequently utilized in a short synthesis of the *cis*-imidazoline small molecule inhibitor (–)-Nutlin-3 (**11**) (Figure 7B). This substance is a potent inhibitor of p53-MDM2 and it is used extensively as a probe of cell biology. Inhibition of the p53-MDM2 protein-protein interaction is a recognized target for the development of anti-cancer drugs and (–)-Nutlin-3 is currently being used in drug development. The synthesis started with the nitro-Mannich reaction leading to **107**, which was obtained in 13:1 dr, 91% *ee*, and nearly quantitative yield. After a single fractional recrystallization, **107** was obtained as a single diastereomer (>200:1) with 97% *ee*. Cobalt-catalyzed reduction of the nitro group and subsequent amidation led to intermediate **108**. Boc deprotection and acylation of the amine with carbonyl diimidazole led to an intermediate isocyanate that was treated with piperazinone **109**. To obtain the desired imidazoline, a chemoselective cyclizative dehydration could be performed using the powerful dehydrating agent phosphonium anhydride, which was formed *in situ* from triphenylphosphine oxide and triflic anhydride (Hendrickson's reagent) (Hendrickson and Hussoin, 1987). The use of the Hendrickson's protocol for reactions of *o*-phenylenediamines ensured that the desired chemoselectivity was achieved in the cyclization of this mixed amide/carbamate substrate. (–)-Nutlin (**11**) was obtained in 88% isolated yield.

Two years later Johnston and coworkers disclosed an improvement on this synthesis which made possible the preparation of (–)-Nutlin on a decagram scale (Davis et al., 2013). The key to the improved protocol was the discovery of a better catalyst, which provided high enantioselectivity at a warmer temperature (−20°C) and low catalyst loadings. Hence, with a loading of 0.5 mol% and **Cat-11** as catalyst, used at −20°C in toluene for 23 h, 23.1 g of the nitro-Mannich product **107** could be obtained with a 90% yield, 200:1 dr and 91% *ee*. After recrystallization, the yield of **107** decreased to 16.0 g, but (–)-Nutlin was obtained with the same 200:1 dr and an *ee* of 99%. More recently these methodologies were applied in an intermittent-flow setup for an automated synthesis of the API (Tsukanov et al., 2016).

Since many pharmaceuticals incorporate fluorine, and half of the existing agrochemicals are also fluorinated molecules, whose syntheses frequently depend on the existence of readily available chiral fluorinated building blocks, the developments of new methods to obtain them is a continuous necessity. In 2018, about 50% of the small molecule drugs approved by the Food and Drug Administration contained fluorine (Szpera et al., 2019). Liu and coworkers developed recently a highly enantioselective aza-Henry reaction of fluoromethylated imines, e.g., **110**, catalyzed by cinchona-derived bifunctional thiourea **Cat-12** (Figure 7C). The fluorinated nitro amines (**111**) produced were ultimately converted (via **112**) into optically pure 4-fluoromethyl-2-imidazolidones, e.g., **113**, after reduction of the nitro group, reaction with triphosgene under basic conditions, and removal of the protecting group with CAN (Li et al., 2019). Compounds **113** may be useful intermediates for the synthesis of fluorinated compounds.

## Oxindoles and Indolenines

**Figure 8:**
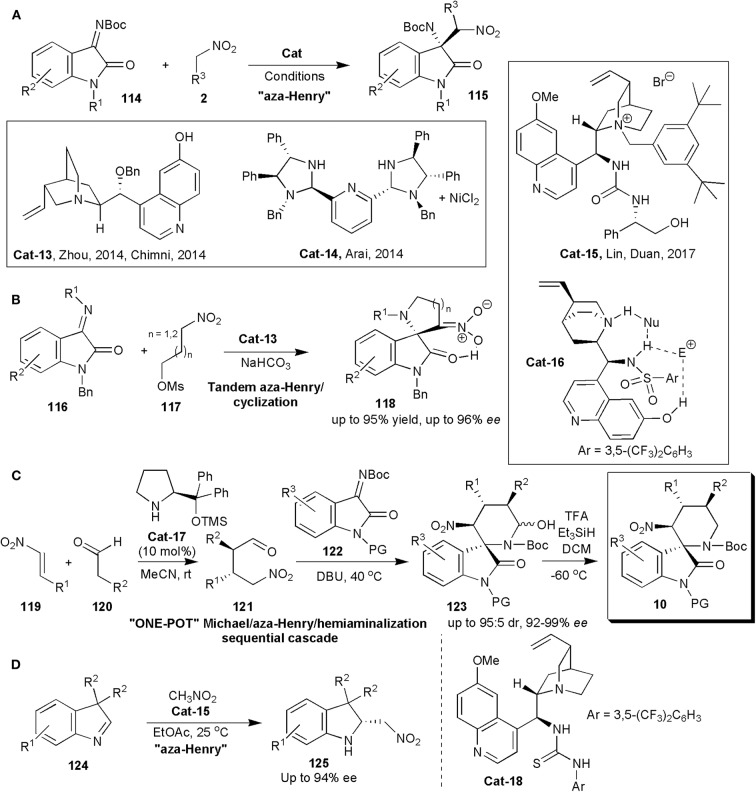
Stereoselective synthesis of oxindoles **115 (A)**, **118 (B)** and **10 (C)**, and of indolenine **125 (D)** via nitro-Mannich reactions.

An oxindole framework is encountered in many biologically active natural products and pharmaceutical substances. This is in fact considered a privileged framework. The 3-subtituted 3-amino-2-oxindole derivatives bearing a tetra-substituted stereocenter are important frameworks in this context, since they are found in several clinical candidates (Chauhan and Chimni, 2013). To construct the quaternary aminocarbon center, isatin-derived ketimines are convenient precursors. In 2014, within a short period of time, three distinct reports were published, in which the quaternary carbon center was created by an enantioselective nitro-Mannich reaction (Figure 8A). Zhou and coworkers showed with one example that in the presence of molecular sieves, quinine-derived **Cat-13** (Figure 8A) could promote the reaction of nitromethane with ketimine **114** (R^1^ = Me, R^2^ = H), affording the product (**115**) in 93% yield and 71% *ee* (Wang et al., 2014). Further developments with this catalyst by Chimni and coworker were published soon after (Kumar et al., 2014). They found that besides the presence of a free amine, a free 6'-OH group on the catalysts had an influence on enantioinduction, since when this group was replaced by OMe, a low *ee* of 27% was obtained. High yields and *ees* up to 89% were obtained in this work in which several ketimines and nitroalkanes were studied.

Arai and coworkers could raise the yields and *ees* even further (Arai et al., 2014). They used an (*S, S*)-diphenyldiamine-derived bis(imidazolidine)-pyridine ligand complexed to Ni (**Cat-14**) (Figure 8A) to catalyze the same reaction. The products were obtained with yields of up to 99% with 95% ee. Lin and Duan relied on phase transfer catalysis, and with bifunctional catalyst **Cat-15** (Figure 8A), obtained the 3-substituted 3-amino-oxindoles in excellent yields (96–99%) and *ees* up to 95% in the nitro-Mannich reaction of isatin-derived *N*-Boc ketimines with nitroalkanes (Liu et al., 2017).

Hajra and Jana used quinine-based trifunctional sulphonamide organocatalyst (**Cat-16**) to promote an asymmetric organocatalytic tandem aza-Henry reaction-cyclization of isatin-derived ketimines (**116**) and nitroalkane-mesylates (**117**) leading to spiro-pyrrolidine/piperidine-oxindoles (**118**) (Figure 8B; Hajra and Jana, 2017). In this case the authors also found that demethylation of the traditional bifunctional catalyst to incorporate an additional hydrogen bonding C6′-OH group was useful, since the OH group played an important role in enantioinduction. Products **118** were obtained exclusively in the enol form, as confirmed by deuterium exchange studies in DMSO-d_6_. The *ees* of the products were in the range 92–96%.

In 2017 Peng, He, Han and coworkers produced pharmacologically interesting piperidine-fused spiro-oxindole derivatives via an asymmetric organocatalytic Michael/aza-Henry/hemiaminalization sequential cascade (Yang et al., 2017). The authors were interested in these substances, since some C3-spirocyclic oxindoles are inhibitors of the MDM2–p53 interaction, and are effective cancer cell proliferation inhibitors. In fact spiro-oxindoles modified at C3 with a rigid nitrogen heterocyclic system have been found to be more effective than oxygen- or sulfur-containing heterocyclic C3-substituted molecules. Some six-membered piperidine- or piperidone-based spiro-oxindole derivatives are known to inhibit the MDM2–p53 interaction with submicromolar IC_50_ values, but only racemic samples had been screened (Yang et al., 2017). The main objective of this study was to synthesize enantiomerically pure substances. The Hayashi–Jørgensen catalyst (**Cat-17**) was effective in catalyzing the initial Michael addition reaction between aldehydes and nitrostyrenes (Figure 8C). The subsequent aza-Henry/hemiaminalization reaction was triggered when the ketamine (**122**) and triethylamine were added to **121** in the same pot at the end of the first reaction. The yields and diastereoselectivities of these reactions were good, and the enantioselectivities excellent, particularly after direct dehydroxylation of the hemiaminal produced with Et_3_SiH and TFA to the more stable products **10**. The absolute configuration of compounds **10** was determined for one by X-ray analysis and the configuration of the others was assigned by analogy. All the compounds showed some activity, although those which were *N*-Boc protected were more active than compounds in which the Boc protecting group on the piperidine nitrogen was removed. The most potent was **10a** (R^1^=H, R^2^=Me, R^3^=4-BrC_6_H_4_, PG=H) which showed the strongest MDM2 inhibition and anti-proliferative effects on breast cancer cells expressing wild-type p53. It inhibited the interaction between MDM2 and p53 peptide with an IC_50_ of 0.91 ± 0.12 μM, and it suppressed proliferation of five breast cancer lines: MCF-7 (IC_50_ 2.5 μM), ZR-75-1 (3.3 μM), BT-474 (35.5 μM), MDA-MB-231 (47.3 μM), and SKBR-3 (41.8 μM). Molecular dynamics simulations also performed suggest that **10a** binds to MDM2 in a manner similar to that of p53. It may induce cell cycle arrest at G0/G1 and it may also cause mitochondrial apoptosis by up-regulating p53 and p21.

In 2019 *Cinchona*-alkaloid-based thiourea bifunctional organocatalyst **Cat-18**, was used by Cheng and coworkers to catalyze an aza-Henry reaction and in this way obtain a series of structurally novel and optically active 2-nitromethyl indolines (**125**) from racemic 3,3-disubstituted 3*H*-indoles (**124**) (Figure 8D; Shao et al., 2019a). The products, which are useful synthetic intermediates, were obtained in useful yields (up to 91%) and good to high enantioselectivities (up to 94% *ee*).

## Quinolizines, Dihydroisoquinolines, and Other Azabicycles

**Figure 9:**
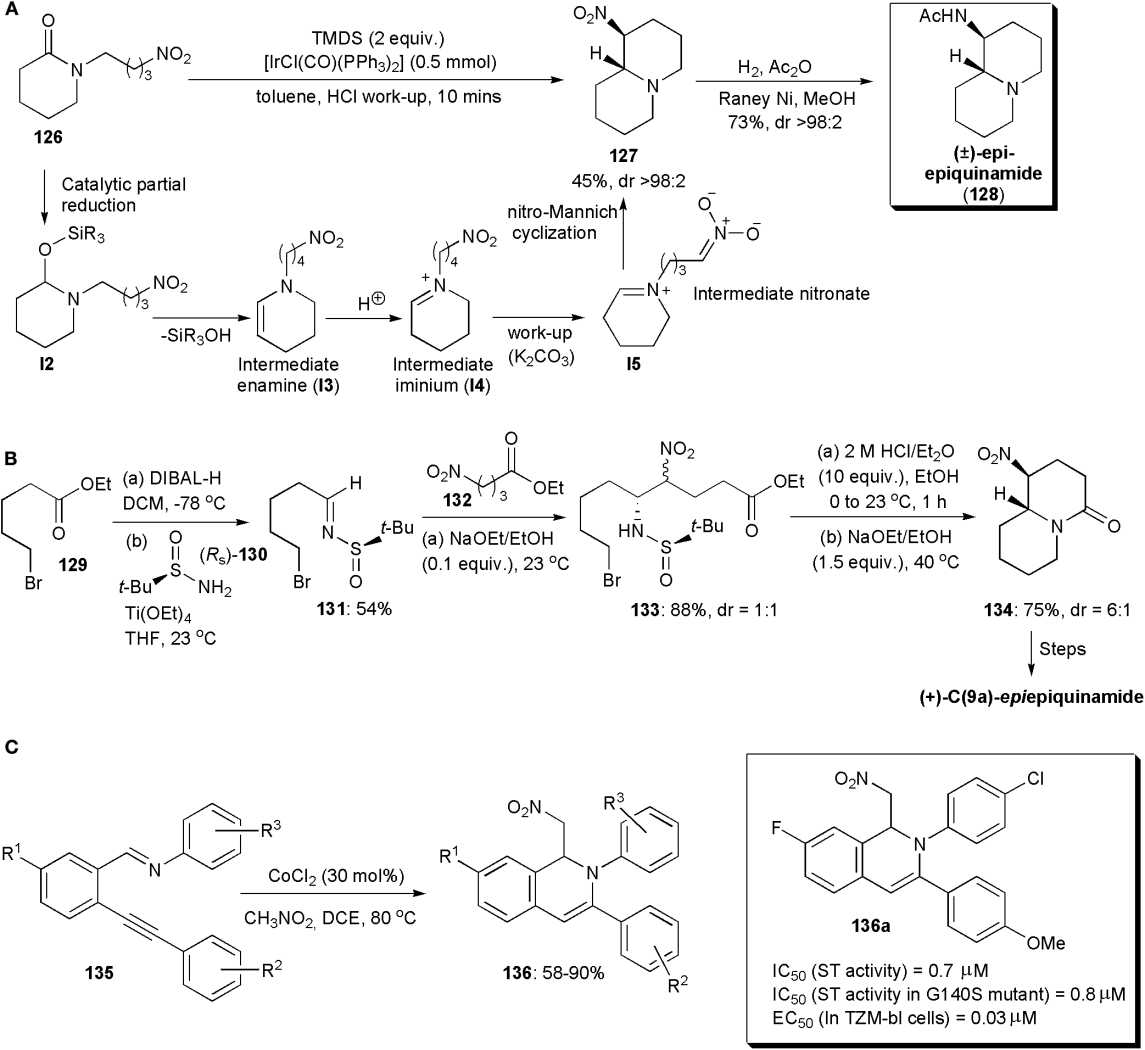
The synthesis of **(A)** (±) -epi-epiquinamide, **(B)** (+) -C(9a)-*epi*epiquinamide and **(C)** isoquinoline **136a**.

A chemoselective reductive nitro-Mannich cyclization of nitro-tethered lactams, e.g., **126**, was developed by Dixon and coworkers in 2015 (Figure 9A; Gregory et al., 2015). The development was based on the fact that in the presence of iridium(I), lactams can be reduced rapidly and chemoselectively to enamines. Vaska's complex was used as catalyst, and the intermediate enamine **I3** produced was converted into an iminium (**I4**) during acidic work-up (HCl, pH 1), which could be detected by ^1^H NMR spectroscopy. During basification with solid K_2_CO_3_, an intermediate nitronate (**I5**) could be formed, which cyclized via a nitro-Mannich reaction yielding bicyclic products, e.g., **127**. The authors showed that the reaction was compatible with a number of substrates, and applied the method also to the synthesis of the natural compound (±)-*epi*-epiquinamide (**128**), which was obtained in a highly diastereoselective manner (Figure 9A). The relative (R^*^, S^*^) configuration was confirmed by comparison with literature data.

Epiquinamide is a quinolizidine alkaloid which has been isolated from the skin extracts of an Ecuadorian poisonous frog, *Epipedobates tricolor*. Initially it was thought to have nicotinic acetylcholine agonistic activity, but this was later disproven (Benlahrech et al., 2018). However there is still considerable interest in the compound, not only because of its interesting structure but also in the hope that it will display other biological activities. In 2018, a stereoselective synthesis of (+)-*C(9a)-epi*epiquinamide was demonstrated by Behloul, Foubelo, Yus and coworkers. A nitro-Mannich reaction was also used in this synthetic route (Figure 9B; Benlahrech et al., 2018). A chiral starting material was employed, (*R*_S_)-*tert*-butanesulfinamide (**130**), which upon reaction with the aldehyde derived from ester **129** gave rise to chiral *N*-*tert*-butanesulfinyl imine **131**, which underwent a high yielding nitro-Mannich reaction with nitroester **132**. The reaction proceeded with almost total facial diastereoselectivity regarding the imine functional group. The other stereogenic center in the product (**133**), i.e., the one bearing the nitro group, was produced as an almost 1:1 mixture of epimers, due to a rapid epimerization which occurred under the basic conditions used, given the acidic character of the proton on that stereocentre. To assemble the bicyclic core of the molecule basic conditions were used again. The *trans*-fused product **134** was obtained preferentially (dr = 6:1), since it is the more stable isomer, with the nitro group occupying an equatorial orientation in a chair-chair conformation. Finally, reduction of the nitro group in **134**, lactam reduction to a bridge trialkylamine derivative, and acetylation of the primary amine yielded the target molecule.

Certain isoquinolines capable of chelating Mg^2+^ ions cause inhibition of the HIV-1 integrase (IN) and/or the HIV-1 reverse transcriptase ribonuclease H domain, e.g., those containing a 2-hydroxyisoquinoline-1,3-(2*H*,4*H*)-dione scaffold (Klumpp et al., 2003). It has been shown that they can inhibit viral replication of HIV-1 in MT-4 cells (Billamboz et al., 2011). Since the HIV-1 virus has high mutation ability, the development of novel and potent INs is the subject of much research. In 2015 Tandom and coworkers developed methods for the synthesis of new 1,2-dihydroisoquinolines, and found in an *in vitro* strand transfer assay, that some of them were potent integrase inhibitors, with IC_50_ values of 0.7–0.8 μM, e.g., **136a** (Figure 9C; Urvashi et al., 2015). In an antiviral assay, one even reduced the level of the p24 viral antigen by 91%, which is comparable to RAL, an FDA approved antiviral drug. Furthermore, the new compounds showed similar strand transfer inhibitory activity in a G140S mutant, which suggests that they can act against resistant strains. The new 1,2-dihydroisoquinolines were synthesized so as to bear different functional groups at the C-1, C-3, C-7, and N-2 positions. Isoquinolines **136** bearing a nitromethyl substituent at C-4, were obtained by a 6-*endo-dig* cyclization/nitro-Mannich reaction catalyzed by cobalt chloride, between non-activated 2-(1-alkynyl) arene carboxaldehyde imines **135** preformed from *o*-alkynyl benzaldehydes and amines, and a nitroalkane (Billamboz et al., 2011). Although stereoselectivity is not an issue in this case, this interesting example has been included to inform the interested reader of recent applications of nitro-Mannich reactions in the synthesis of biologically active substances.

**Figure 10:**
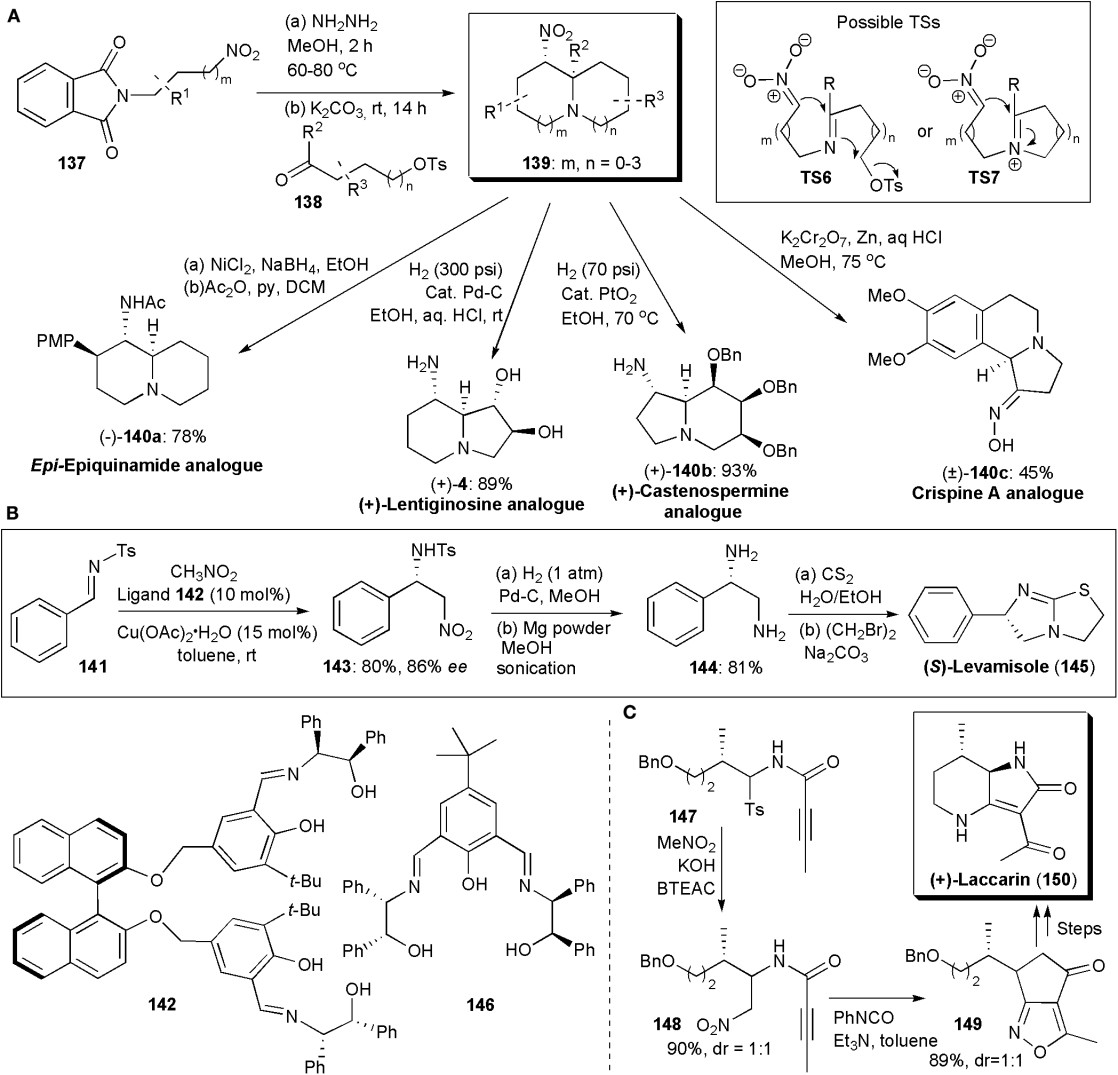
The synthesis of azabicyclic APIs or intermediates via nitro-Mannich reactions: **(A)** diversity in the synthesis of azabicycles obtained via a one-pot cascade nitro-Mannich/alkylation cascade reaction; **(B)** the synthesis of (*S*)-Levamisole; **(C)** the synthesis of (+)-Laccarin.

Many biologically active substances have some kind of azabicyclic skeleton, as for example, epi-epiquinamide shown above (Figures 9A,B). Hence there have been many synthetic approaches to obtain these structural units. Disadee and Ruchirawat developed recently a one-pot cascade nitro-Mannich/alkylation reaction which provides azabicycles in yields up to 81% and an isomeric ratio of 62:1 (Figure 10A; Disadee and Ruchirawata, 2018). The method was shown to be versatile and to give direct access to various biologically active substances. In these procedures, 3-nitro-1-propylamine generated *in situ* from the corresponding phthalimide-protected nitroamine is allowed to react with a suitable aldehyde. The use of conditions that favor nitronate formation and cyclization, while avoiding aldehyde self-cyclization and isomerization, are crucial to the success of this procedure. Potassium carbonate was found to be the best base, which not only made the desired reaction possible but facilitated work-up, since several byproducts, e.g., hydrazide and tosylate, could be easily separated as potassium salts by normal filtration and it could also act as a desiccant removing water from the solvent to promote imine formation. Products **139** with various ring sizes and substitution patterns could be obtained. The authors showed the applicability of the method to the stereoselective synthesis of analogs of several alkaloids with diverse biologically activities (Figure 10A; Harit and Ramesh, 2016).

Besides epiquinamide analog (–)-**140a**, an analog of the iminosugar *trans*-1,2-dihydroxyindolizidine (+)-lentiginosine [(+)-**4**], a potent inhibitor of amyloglucosidase, and a good inhibitor of Hsp90, (Pastuszak et al., 1990), was also obtained. The non-natural enantiomer of lentiginosine, (+)-lentiginosine, displays anti-tumor activities. This class of compounds has been the subject of several studies. Iminosugar *castanospermine* is known to inhibit all forms of α- and β-glucosidases. Besides that, it is also a potent inhibitor of Dengue virus infection *in vitro* and *in vivo* [34] and it has been studied as an anti-cancer agent. Compound (+)–**140b** is an analog of this substance. Crispine A, with a tetrahydroisoquinoline unit fused to a third ring, is an alkaloid isolated from *Carduus crispus*. Traditionally, *C. crispus* has been used in Chinese folk medicine to treat colds, stomach-ache and rheumatism. Some *C. crispus* extracts have also been reported to be significantly cytotoxic against SKOV3, HeLa and KB human cancer cell lines (Rotte et al., 2013). Compound (±)-**140c** is an analog of Crispine A.

The synthesis of imidazothiazole-derivative Levamisole, an anthelminthic agent, has been the subject of a few studies. In a formal synthesis of this molecule published in 2014 by Kureshy and coworkers, an enantioselective copper-catalyzed aza-Henry reaction is used to establish the chiral center in the molecule (Choudhary et al., 2014). Amino alcohol-based ligands, particularly **142**, were found to promote the reaction between aliphatic *N*-tosylaldimines and aromatic *N*-benzenesulfonamide aldimines and nitromethane, providing products in high yields and very high enantio- and diastereoselectivities. Figure 10B shows the synthetic route used, via **143** and **144**, which allowed the preparation of 1 g of Levamisole. In addition, the catalyst, Cu(II)-ligand **142**, could be recovered and recycled up to five times without losses in yield or selectivity. One year later the Kureshy group developed another set of ligands for copper-catalyzed aza-Henry reactions, finding that amino alcohol **146** provided the best results, 96% *ee* and a good yield (Choudhary et al., 2015). The Cu-**142** catalyst could also be recycled five times and it was used for the synthesis of Levamisole by to the same synthetic scheme.

(+)-Laccarin (**150**) is an acylpyrrolidinone which has been isolated from the mushroom *Laccaria vinaceoavellanea* and exhibits cyclic AMP phosphodiesterase inhibitory activity (Katsuta et al., 2018). Katsuta and coworkers developed a synthetic approach to this substance using a pre-synthesized 3:2 diastereomeric mixture of α-amidosulfone **147**, which when subjected to a nitro-Mannich reaction with nitromethane yielded a 1:1 mixture of **148** (Figure 10C). An intramolecular 1,3-dipolar cycloaddition reaction afforded **149**, from which laccarin could be obtained in three additional synthetic steps, in overall moderate yield.

## Larger Fused Ring Systems Containing A Shared Nitrogen

**Figure 11:**
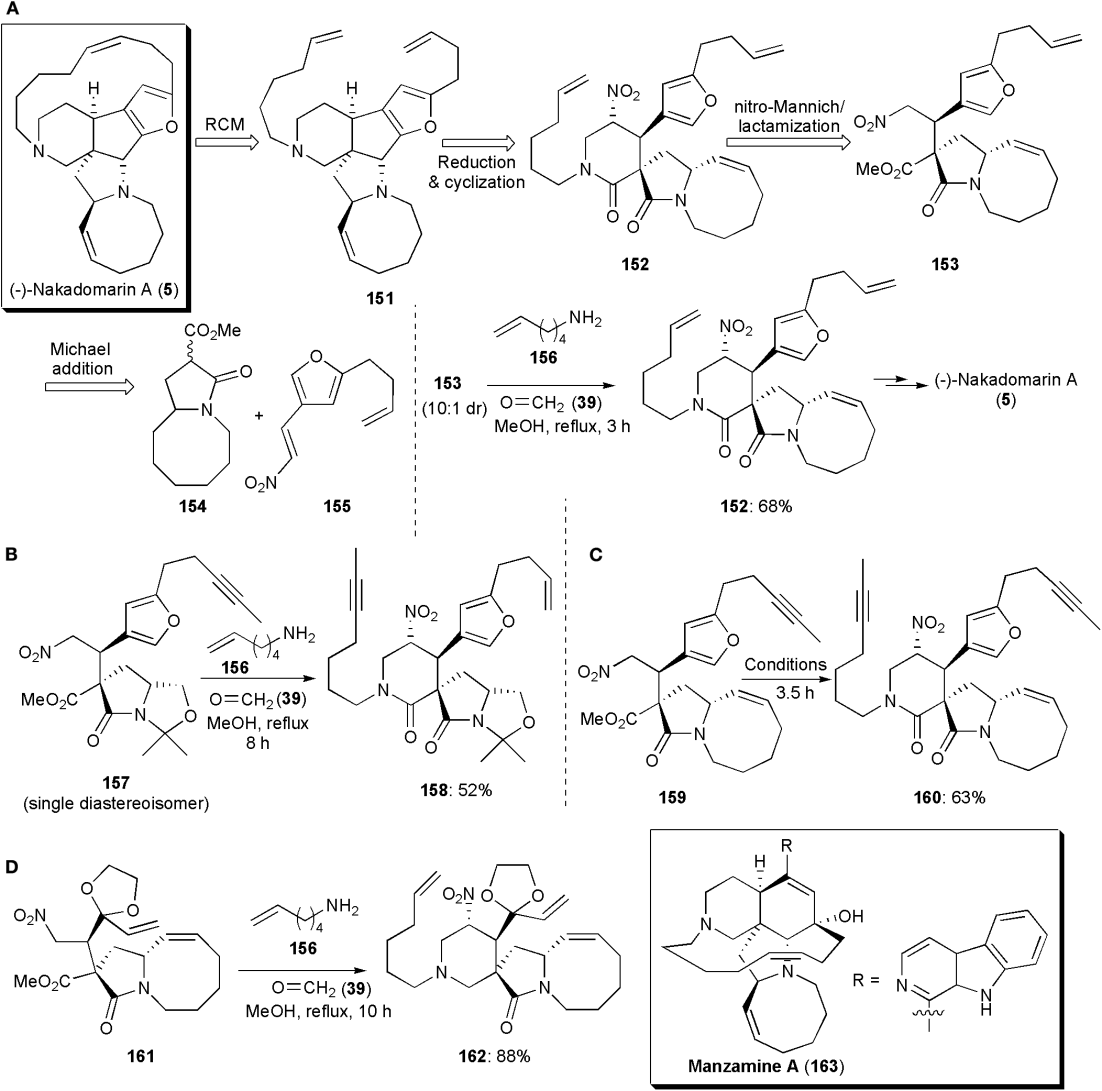
The application of a nitro-Mannich/lactamization cascade reaction in the synthesis of large-fused ring systems: **(A–C)** different approaches to (–)-Nakadomarin A; **(D)** the synthesis of intermediate **162** toward Manzamine A.

Interesting large ring systems whose syntheses have comprised a nitro-Mannich reaction are some alkaloids of the manzamine family. Nakadomarin A (**5**) was one of the first known, and it was isolated by Kobayashi from an Okinawan sea sponge, *Amphimedon* sp., in 1997 (Kobayashi et al., 1997). It displays cytotoxic activity against murine lymphoma L1210 cells (IC_50_ = 1.3 μg/mL), inhibition of cyclin dependent kinase 4 (IC_50_ = 9.9 μg/mL) and exhibits antimicrobial activity against the fungus *Trichophyton mentagrophytes* (MIC = 23 μg/mL) and the Gram-positive bacterium *Corynebacterium xerosis* (MIC = 11 μg/mL). These important properties, combined with its elaborate hexacyclic ring system, an 8/5/5/5/15/6 ring system with four stereogenic centers, have arisen much interest not only in the development of synthetic routes to obtain the alkaloid, but also others of the manzamine family. A method developed for total synthesis of (-)-Nakadomarin A reported by Dixon and coworkers in 2009, included a nitro-Mannich reaction, which was utilized to assemble the six-membered ring as shown in its retrosynthetic scheme (via intermediates **151**–**155**) (Figure 11A; Jakubec et al., 2009). In the actual synthesis, an imine formed *in situ* from commercial hex-5-enamine (**156**) and formaldehyde (**39**) reacted with nitro ester **153** (10:1 dr), via a diastereoselective multicomponent nitro-Mannich/lactamization cascade reaction, to afford the desired pyranone ring. Intermediate **152** was obtained with a good yield of 68%. In the cascade reaction, conditions similar to those established previously by the group for the synthesis of paroxetine and for other piperidinones, were used (Hynes et al., 2008; Jakubec et al., 2008). In later years the Dixon group published further refinements on this synthesis. Since the metathesis reaction in which the 15-membered ring of (-)-Nakadomarin A was constructed led to the formation of E/Z isomeric mixtures, a new route (Figure 11B) was developed aiming to improve this step, and an alkyne RCM was chosen as the key macrocyclic ring-forming step (Kyle et al., 2011). A nitro-Mannich/lactamization cascade was also used to assemble the six-membered ring (Scheme 10b). In fact, this approach turned out to be very versatile, and it could be used in a few distinct total synthetic routes to this alkaloid. In this route, which proceeds via the reaction of **157**–**158**, the formation of the eight-membered ring, also by ring-closing metathesis, was the last step.

A shorter route, a 13-step highly stereoselective synthesis, was also reported by the Dixon group in the same year (Figure 11B; Jakubec et al., 2011). The approach used also relied on a bifunctional organocatalyst-controlled Michael addition, a nitro-Mannich/lactamization cascade, an alkyne ring-closing metathesis/syn-reduction, and furan/iminium ion cyclization/reduction as key steps. The piperidinone ring-forming step (**159**–**160**), was achieved under conditions similar to those already reported, affording the product in 63% yield (Figure 11C). In this third generation route, the synthesis is concluded by a novel furan/iminium ion cyclization step, from which the *cis*-fused 5,5-ring system is obtained with high diastereoselectivity. In each one of these synthetic sequences, the nitro group, which is used in the initial steps first to activate a highly diastereoselective Michael addition reaction, and subsequently to bring about the nitro-Mannich/lactamization cascade which gives rise to the six-membered ring of (-)-Nakadomarin A, is finally removed during the synthetic sequence through AIBN-Bu_3_SnH reduction. The carbonyl group of the piperidinone ring is reduced in the first and third synthetic approaches with LiAlH_4_. In the second generation synthesis, a selective reduction to the piperidine is achieved with DIBAL-H.

Manzamine A (**163**) is a complex natural product, also a marine alkaloid isolated from a sponge in the Okinawa Sea by Sakai et al. (1986). It displays a range of potent biological properties, including insecticidal, anti-bacterial, anti-inflammatory, anti-cancer, and anti-malarial activity. This alkaloid includes in its structure a pentacyclic core comprising 6-, 6-, 5-, 13-, and 8-membered rings, with five stereocenters. Attached to the 6-membered carbocyclic ring is a β-carboline heteroaromatic ring system. The development of synthetic routes to obtain this substance has also arisen considerable interest. One of them, from the Dixon group, includes a nitro-Mannich reaction to construct the 6-membered heterocyclic ring (Jakubec et al., 2012b). In the synthesis of Manzamine A, this ring is built near the start of the synthetic sequence. The nitro-Mannich/lactamization cascade employed in the synthesis of (-)-Nakadomarin A also worked well in this case. Reaction of nitro ester **161**, used as a single diastereoisomer after isolation by column chromatography, with formaldehyde and hex-5-en-1-amine in refluxing methanol, afforded the 4-nitro-piperidin-2-one **162** as a single diastereoisomer in high yield (**Figure 11D**). This synthetic route toward Manzamine A, with its 18-steps in the longest linear sequence, turned out to be the shortest reported until then.

**Figure 12:**
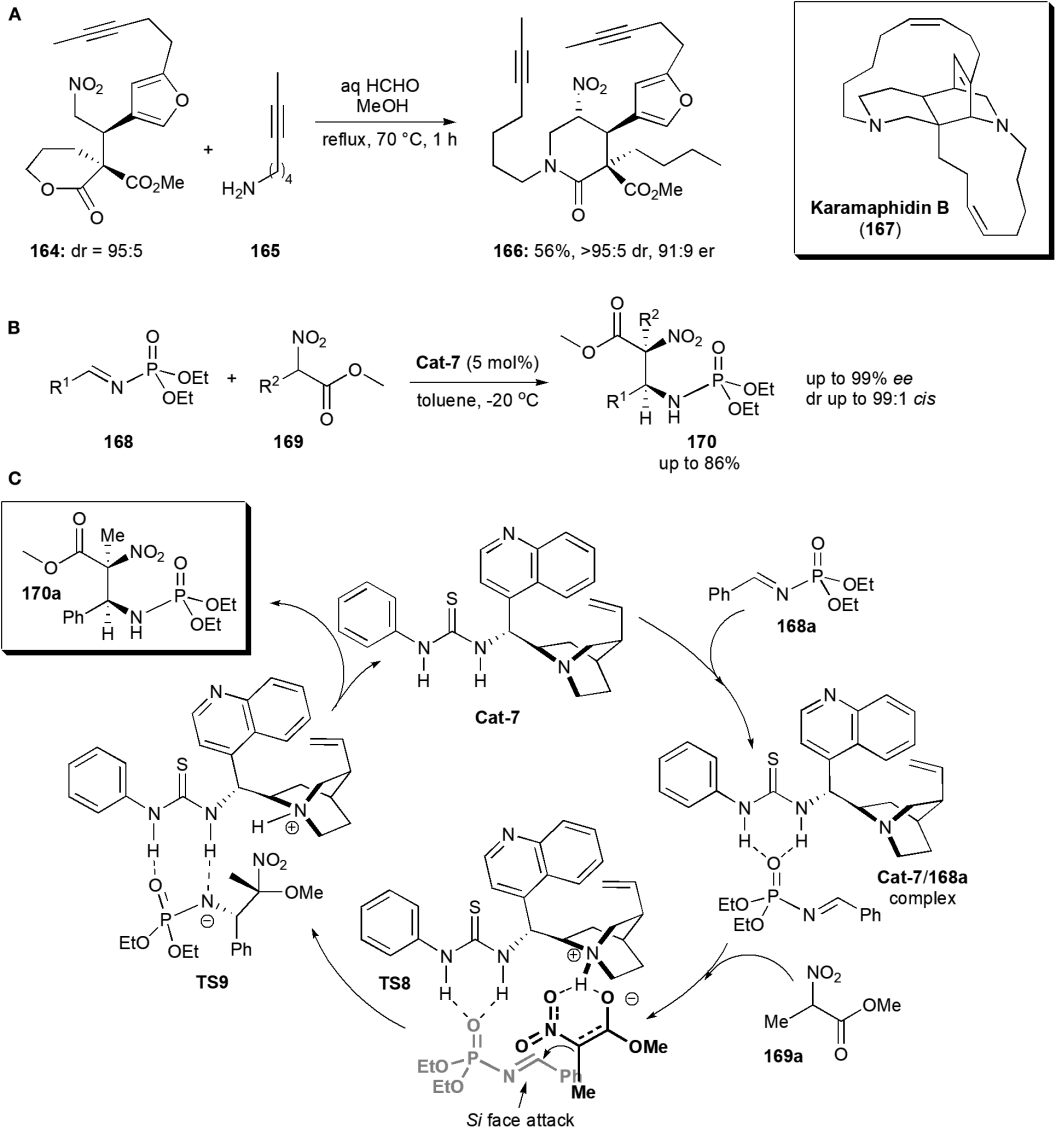
**(A)** The synthesis of intermediate **166** toward the alkaloid Karamaphidin B; **(B)** synthesis of a phosphoramidate derivative via a nitro-Mannich reaction; **(C)** the mechanism proposed for the synthesis of phosphoramidates **170**, exemplified with **170a** (Fan et al., 2013).

Keramaphidin B (**167**) is another alkaloid isolated from the Okinawan marine sponge *Amphimedon* sp., which was also shown to be cytotoxic against cancer cells, e.g., the KB human epidermoid carcinoma cells (IC_50_ 0.28 μg/mL) and P388 murine leukemia cells (IC_50_ 0.28 μg/mL) (Kobayashi et al., 1994). It is also a member of the manzamine family of alkaloids, and it contains an intricate structure comprising a 6,6,6,11,13 pentacycle possessing 4 stereogenic centers including one quaternary center. Dixon and coworkers reported an approach to the synthesis of this alkaloid in 2016 (Jakubec et al., 2016). The stereochemistry of the molecule was established in an early step, a Michael addition reaction of a δ-valerolactone-derived pronucleophile to a substituted furanyl nitroolefin catalyzed by a bifunctional cinchonine-derived thiourea. The product, nitro ester **164**, obtained in high yields as a 95:5 mixture of diastereoisomers, was subsequently exposed to the nitro-Mannich lactamisation cascade used previously (cf Scheme 3c), in the presence of **165**, to afford piperidinone **166** in 56% yield, and very high dr and *ee* (Figure 12A). The cascade reaction was chemoselective, favoring amine attack at the more reactive δ-lactone carbonyl carbon of **164**, rather than at the methyl ester.

Having fulfilled its role, the nitro group was removed by a traceless reductive cleavage using modified Ono's conditions, with tributyltin hydride and AIBN. An important intermediate toward the synthesis of keramaphidin could thus be synthesized from piperidinone **166** in a few more steps.

## Aminophosphonic and Amino Acid Derivatives

These substances are frequently used as intermediates in the synthesis of biologically active compounds. Since most of works reported concentrate mainly on method development rather than applications to specific biologically active targets, only a few representative recent examples are included in this review. There are many phosphoramidate derivatives which exhibit important biological activities, including anticancer and antiviral (Fan et al., 2013). Some have found applications in medicinal chemistry. In 2013 Miao and coworkers reported a procedure to obtain phosphoramidates bearing 1,2-diamine moieties (Fan et al., 2013). Substances with this structure are relatively rare. The compounds described also had adjacent quaternary and tertiary chiral centers. The presence of a quaternary stereocenter in a highly substituted phosphoramidite unit, is expected to interact with certain proteases and resist proteolytic degradation, in a similar manner to that of α-quaternary phosphonates (Faisca Phillips, 2014, 2016, 2019). The method entailed an asymmetric nitro-Mannich reaction of *N*-phosphoryl imines **168** with α-substituted nitroacetates **169** (Figure 12B). Cinchona alkaloid thioureas were used as organocatalysts, and afforded the corresponding β-nitro ethylphosphoramidates **170** in high yield (up to 86%) and high enantiostereoselectivity (up to 99% ee) and diastereoselectivity (up to 99:1, *anti*-selectivity). A mechanism was proposed, based on ^31^P NMR observations (Figure 12C). Hydrogen bond formation between the phosphoryl oxygen atoms and the nitrogen of the *N*-phosphoryl imine and the catalyst, as in complex **168a**, increase the electrophilicity of the imine. The nitronate, created by the action of the Brønsted basic catalyst on the nitroalkane, is brought close to the imine also through complexation with the catalyst in an orientation favoring *Si* face attack on the imine from the *Si* face of the nitronate, as shown in **TS8** and thus producing an (*S,S*)-configured product, which is released from the catalyst upon proton transfer to the negatively charged amine in **TS9**.

**Figure 13:**
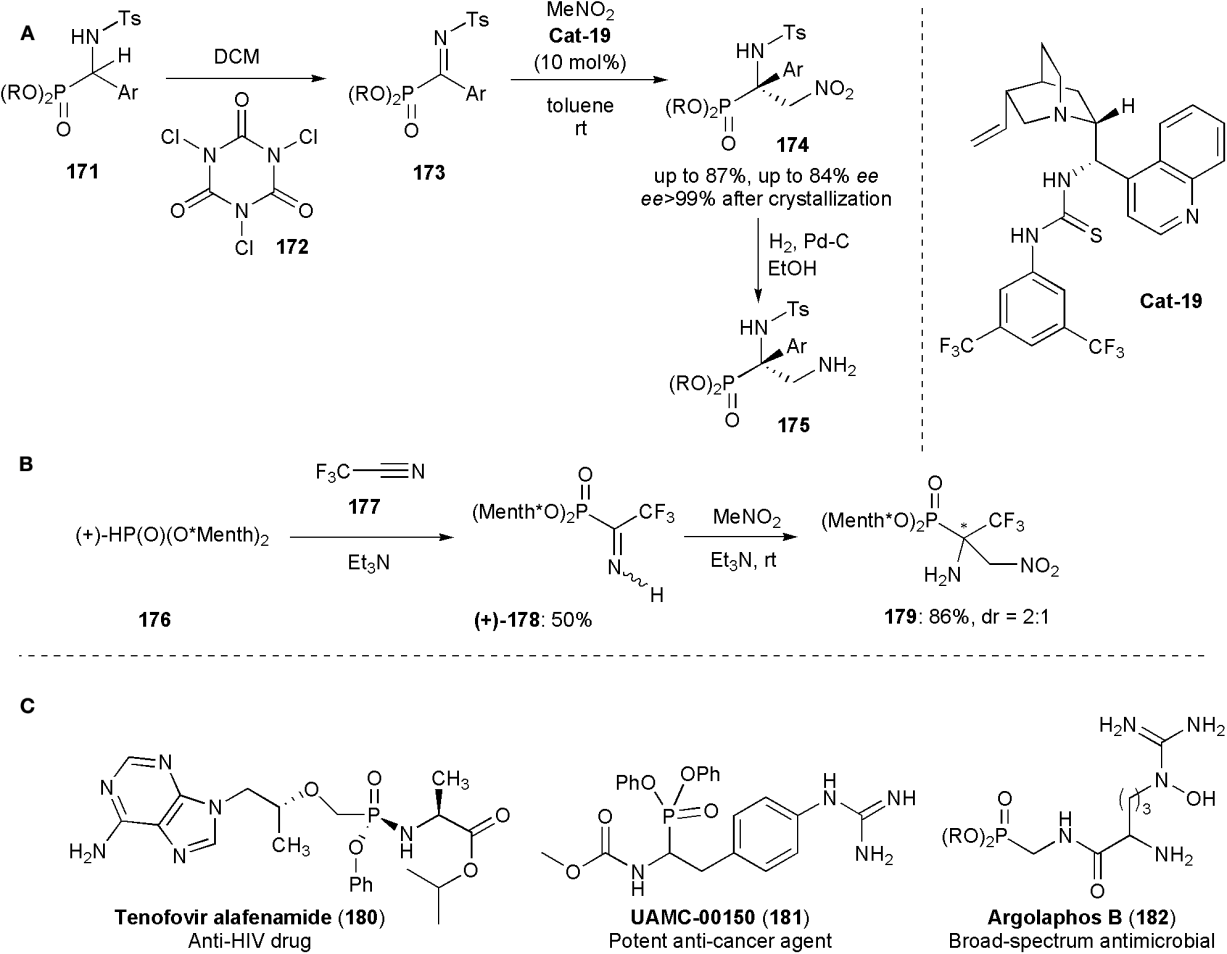
**(A,B)** Synthesis of aminophosphonic acid derivatives **175** and **179** via nitro-Mannich reactions; **(C)** Examples of biologically active phosphoramidate and aminophosphonate substances including the anti-HIV drug Tenofovir alafenamide.

Functionalized tetrasubstituted α-aminophosphonates were obtained by Palacios and coworkers by a nitro Mannich reaction from phosphorylated ketimines **173**, prepared from **171**, and nitromethane (Vicario et al., 2015). Due to their analogy to α-amino acids, α-aminophosphonic acid derivatives have found numerous applications in medicinal and pharmaceutical sciences. The biological activity of these substances depends strongly their absolute configuration (Mucha et al., 2011), but of the methods known until then only a few were effective for the enantioselective synthesis of tetrasubstituted α-aminophosphonates. In this approach, a bifunctional cinchona alkaloid thiourea (**Cat-19**) was used as catalyst (Figure 13A). The tetrasubstituted α-amino-β-nitro-phosphonates **174** were obtained in *ees* up to 84% and were subsequently converted by catalytic hydrogenation into α,β-diaminophosphonates **175**.

Onys'ko and coworkers reported earlier this year the synthesis of α-trifluoromethylated α-aminophosphonic acid derivatives (Rassukana et al., 2019). Optically pure (+)- or (–)-dimenthyl phosphite were reacted with trifluoroacetonitrile to yield *O,O*-dimenthyl α-iminotrifluoroethylphosphonates which were shown to be useful intermediates for the synthesis of a variety of substances, including chiral *N*-tosylated α-amino-β-nitrophosphonates via an aza-Henry reaction. This was the first time that an aza-Henry reaction was used for the synthesis of compounds incorporating a fluoroalkylated-α-aminophosphonate moiety and these were also the first examples reported of chiral fluoroalkylated β-nitro-α-aminophosphonates. The reaction afforded products in high yields, e.g., **179** (86%), but low diastereoselectivity (2:1) (Figure 13B). The *N*-unprotected iminophosphonates (**178**) used in this work were stable solids which could be isolated by column chromatography in 50% yields. In solution they exist as an equilibrium mixture of *Z-* and *E*-isomers. Their NMR spectrum showed that the more sterically hindered Z-configuration is the thermodynamically preferred one. The capability of producing and using unprotected iminophosphosphonates directly is very promising for future developments, because protecting group removal in functionalized phosphonates, particularly when electron-withdrawing groups are present, is often accompanied by cleavage of the C–P bond (Rassukana et al., 2019).

In the syntheses of all these phosphonic acid derivatives, the nitro group which participates in the nitro-Mannich reaction is kept, and it is eventually reduced to an amine. Figure 13C shows examples of a medicinally relevant phosphoramidate (**180**) and phosphonates (**181–182**) bearing alpha-amino substituents (Faisca Phillips, 2019). Argalaphos B has been recently isolated from a spent culture of *S. monomycin* NRRL 24309. It was found through a project on genome mining.

**Figure 14:**
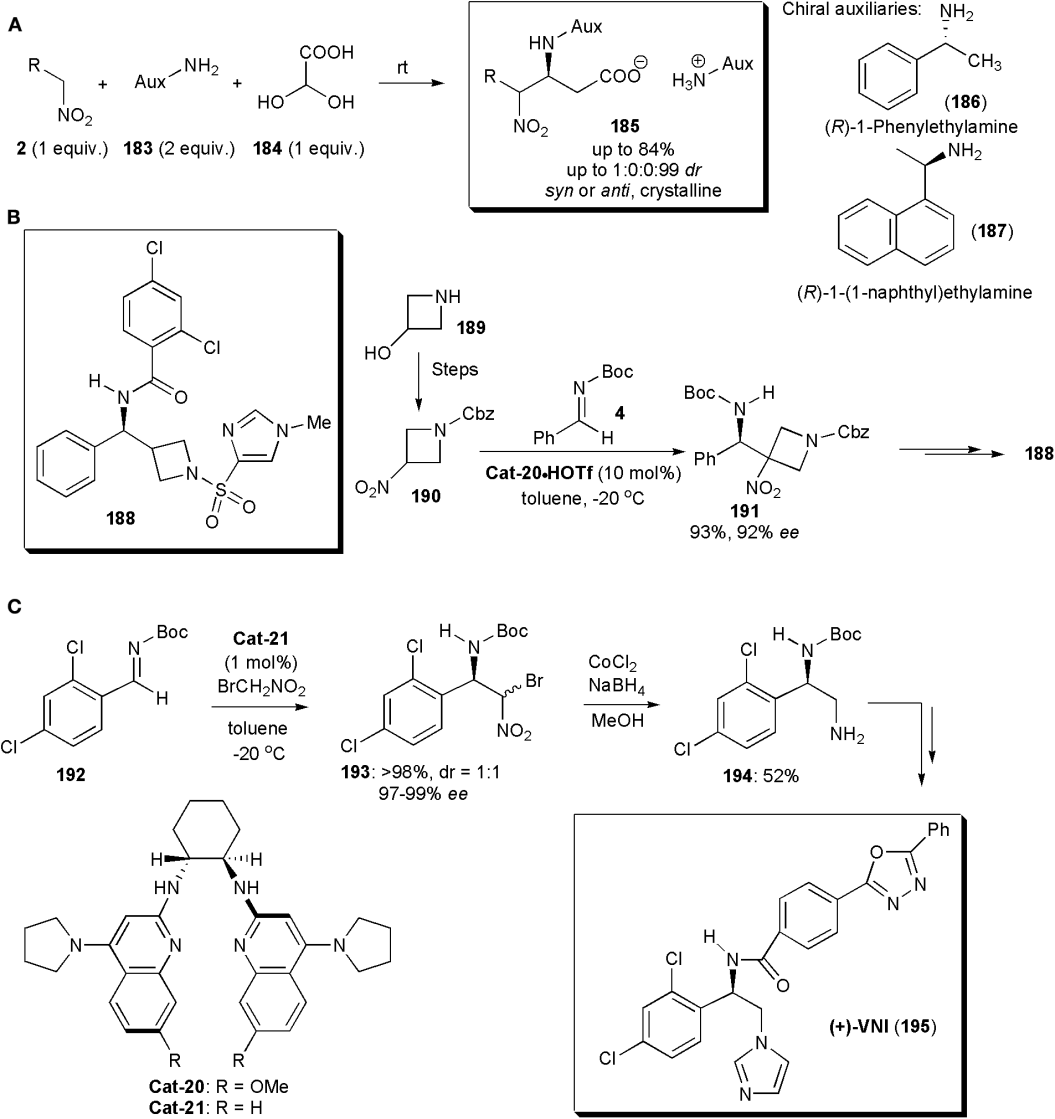
Stereoselective syntheses of **(A)** β-nitro-α-amino carboxylic acid **185**, **(B)** GlyT1 inhibitor **188** and **(C)** the anti-*T. cruzi* (+)-VNI.

Among the recent applications of nitro-Mannich reactions in the synthesis of modified amino acids, an interesting recent example is the stereoselective synthesis of β-nitro-α-amino carboxylic acids, reported by the group of Jakubec (Figure 14A; Marčeková et al., 2019). Aza-Henry reactions of aldimines, yielding either *anti*- or the more rarely reported *syn*-configured products were performed leading to products in very high yields and diastereoselectivities. The necessary imines were generated *in situ* and the presence of a chiral auxiliary in the primary amine used initially as starting material ensures that the reaction proceeds in a stereoselective manner. The products **185** are salts, and their crystallization controls the diastereoselectivity. Indeed, this is a good example of a crystallization-induced diastereoselective transformation. The optimum chiral auxiliary was selected experimentally and it turned out that (*R*)-1-phenylethylamine (**186**), in combination with *i*PrOH as solvent, provided the best results. In some instances (*R*)-1-(1-naphthyl)ethylamine (**187**) was superior. The auxiliary could be subsequently removed by hydrogenation on Pd-C to yield the free amino acid. Another useful feature is the fact that the nitroalkane can be used in equimolar amounts, whereas in the majority of processes reported a large excess is used (Marčeková et al., 2019). This method has broad scope, and it even worked well with conformationally constrained substrates, including sterically demanding α,α-disubstituted nitro compounds. The products **185** were also obtained in very high stereochemical purities and good yields in these cases.

## Miscellaneous Reactions

Besides the major classes of compounds already mentioned, the nitro-Mannich reaction has applications in the synthesis of many other types of substances. One example is azetidine **188**, a potent GlyT1 inhibitor (IC_50_ 29 pM) with potential for the treatment of schizophrenia (Davis et al., 2012). A key step in the synthesis of this substance, by Johnston and coworkers, is a nitro-Henry reaction between a 3-nitroazetidine (**190**) obtained from a 3-hydroxyazetidine (**189**) and an *N*-Boc imine (**15**) (Figure 14B). This reaction afforded a very high yield and *ee* of the desired product **191** when performed in the presence of the triflic acid salts of chiral bisamidine catalysts. Very effective was **Cat-20·**HOTf, which acting as a chiral Brønsted base, deprotonated the nitroalkane creating a chiral nitronate that added to imine **15** delivering the product in a highly enantioselective manner. In this synthetic route, the nitro group is only used to bring about the nitro-Mannich reaction, and it is subsequently removed with ^n^Bu_3_SnH/AIBN. This synthesis is also a rare example of an addition of a secondary nitroalkane to an imine and it was also the first enantioselective synthesis of this GlyT1 inhibitor.

VNI (**195**) is a potent inhibitor of trypanosomal sterol 14α-demethylase (CYP51), disrupting membrane formation in *T. cruzi*, the parasite responsible for Chagas' disease, which affects more than 8 million people throughout the Americas. VNI has already been shown to cure mice infected with *T. cruzi* in both acute and chronic stages of infection, and it could be an alternative to posaconazole, an antifungal drug currently undergoing trials to treat this disease, which has the disadvantage of being very costly. An enantioselective synthesis of VNI, developed by the Johnston's group, relied on a nitro-Mannich reaction to establish the chiral center, and it could even be performed on a 10 g scale (Figure 14C; Dobish et al., 2012). The chiral bisamidine catalyst used was crucial in this process. **Cat-21**, acting as a chiral Brønsted base, deprotonated the nitroalkane creating a chiral nitronate, which added to imine **192** to deliver product **193** as a 1:1 mixture of diastereoisomers, each in 97–99% *ee*. This was in fact not a problem since, because compounds **193** are homochiral at the benzylic position, upon reduction a single diamine (**194**) was obtained. Catalyst **Cat-21** proved to be very active in this transformation (approximately 100 turnovers) with the advantage that it could be recycled without loss of activity.

**Figure 15:**
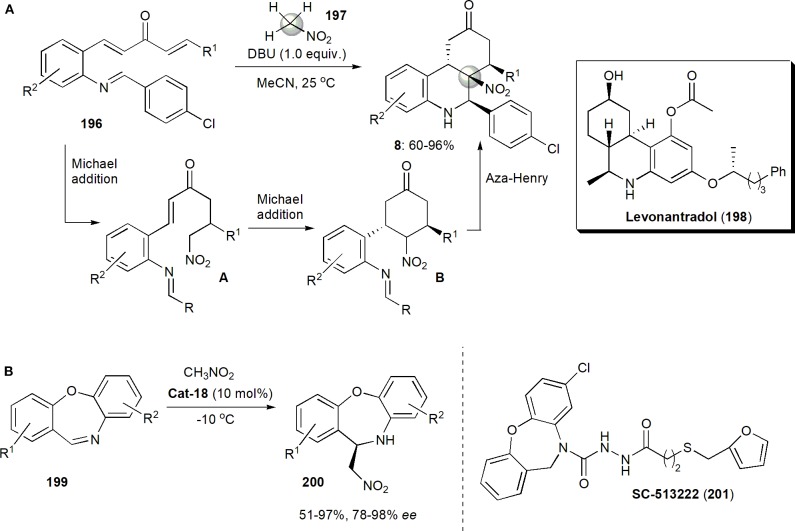
Synthesis of **(A)** the psychoactive Levonantradol and **(B)** oxazepine **200**.

Lu, Xi and coworkers developed recently a procedure to synthesize hexahydrophenanthridin-9(5*H*)-ones (Figure 15A; Feng et al., 2018). The tricyclic core of these substances is often found in many natural products or synthetic molecules which are biologically active. This was achieved in a one pot process, by a stereoselective domino double Michael addition/intramolecular aza-Henry reaction in which nitromethane plays the interesting role of triple nucleophile, forming three C–C bonds. Another pathway is also possible for this reaction, a domino Michael addition/aza-Henry/Michael addition. The first possibility is illustrated in the Figure 15A. A base-promoted intermolecular Michael addition, in which nitromethane attacks the less hindered enone moiety of **196**, generating intermediate **A**, takes place initially. This is followed by an intramolecular Michael addition in the presence of base, which takes place stereoselectively at the less hindered face of the double bond and generates the cyclohexanone ring **B**, and an intramolecular aza-Henry reaction which generates the final product **8**. Reactions in which the 4-chlorophenyl ring was substituted either by *t*-Bu, or 2-thionyl or 2-furyl, afforded mixtures of two diastereoisomers each. In all the other cases the reactions were highly diastereoselective and a single diastereoisomer was produced. The structures of the products were confirmed by X-ray crystallography.

The core tricyclic ring of compounds **8** is analogous to that of levonantradol (**199**), a substance which mimics delta-9-tetrahydrocannabinol (THC), the principal psychoactive ingredient in cannabis, but is 30 times more potent than THC in activating the CB1 and CB2 cannabinoid receptors. Levonantradol also possesses analgesic and antiemetic properties (Sheshenev et al., 2013) and it is 9–14 times more potent than morphine. This substance was once considered a clinical candidate, but it turned out to be too toxic. However, it still remains a tool in the study of the pharmacology of cannabinoid compounds.

Organocatalytic aza-Henry reactions are usually performed with electron-deficient acyclic imines, which are used as electrophiles. Reactions performed with cyclic imines are rare and the first was reported only in 2011, by the group of Wang. It was the synthesis of DPC 083 shown in Figure 6C. The imines were highly reactive. Unactivated cyclic imines are more problematic, and in the only single report known, with reactions between 3,4-dihydroisoquinolines and nitromethane, by Todd and coworkers (Ahamed et al., 2010), the best *ees* obtained were only up to 70%, although the yields were high. In addition the aza-Henry intermediates had to be acylated *in situ*, to overcome the reversibility of the reaction and drs were only moderate. In 2019 Cheng and coworkers studied for the first time organocatalytic asymmetric aza-Henry reactions of dibenzo[*b, f* ][1,4]oxazepines **199** as a representative class of unactivated seven-membered cyclic imines (Shao et al., 2019b). The oxazepine skeleton is found in a number of pharmaceutical substances but up to this report substances containing this structural unit had never been used in aza-Henry reactions. One example is 8-chlorodibenz[b,f][1,4]oxazepine-10(11*H*)-carboxylic acid, 2-[3-[2-(furanylmethyl)thio]-1-oxopropyl]hydrazide (SC-51322), which is a potent PGE_2_ antagonist and analgesic. Cheng's reaction (Figure 15B) afforded high yields of products **134** in very high yields, when quinine-derived thiourea **Cat-18** was used as catalyst. The reaction could even be performed on a gram-scale. The products could be converted into valuable diamines with no loss in enantiopurity.

## Conclusions

Although the first stereoselective nitro-Mannich reaction was developed only 20 years ago, many developments have taken place since then. There are nowadays several applications in the synthesis of APIs and other biologically active substances, which include highly diastereoselective and enantioselective processes. The versatility of the nitro group, which can be either kept or converted into an amine, a ketone, or simply be removed, is in part responsible for the popularity of the reaction in target-oriented synthesis. Hence a wide range of products, with diverse structural composition, have been reported: five-, six-, and seven-membered nitrogen heterocyclic compounds, azabicyclic substances and complex alkaloids, to name a few. The fact that many metal-based or organocatalysts compatible with a large variety of functional groups have been developed since has contributed to broaden the scope of the nitro-Mannich reaction. We hope that the examples highlighted will be an inspiration for further developments in this area.

## Author Contributions

All authors listed have made a substantial, direct and intellectual contribution to the work, and approved it for publication.

### Conflict of Interest

The authors declare that the research was conducted in the absence of any commercial or financial relationships that could be construed as a potential conflict of interest.

## Abbreviations

AIBN: Azobisisobutyronitrile
BTEAC: Benzyltriethylammonium chloride
Boc: *Tert*-butyloxycarbonyl
CAN: Ceric ammonium nitrate
Cbz: Benzyl chloroformate
DBU: 1,8-Diazabicyclo[5.4.0]undec-7-ene
DCE: Dichloroethane
DIBAL-H: Diisobutylaluminium hydride
DMAP: 4-Dimethylaminopyridine
EDC: 1-Ethyl-3-(3-dimethylaminopropyl)carbodiimide
Fmoc: Fluorenylmethoxycarbonyl
HOBT: Hydroxybenzotriazole
MCPBA: Meta-chloroperbenzoic acid
Py: Pyridine
TBAF: Tetra-*n*-butylammonium fluoride
TBE: *Tert*-butyl methyl ether
TBSCl: *Tert*-Butyldimethylsilyl chloride
TEA: Triethylamine
TFA: Trifluoro acetic acid
TMAF: Tetramethylammonium fluoride
TMDS: Tetramethyldisiloxane.
